# RAS70 peptide targets multiforme glioblastoma by binding to the plasma membrane heat shock protein HSP70

**DOI:** 10.3389/fonc.2025.1543657

**Published:** 2025-03-24

**Authors:** Maxim Shevtsov, Natalia Yudintceva, Danila Bobkov, Ruslana Likhomanova, Anastasiya Nechaeva, Elena Mikhailova, Elena Oganesyan, Viacheslav Fedorov, Andrey Kurkin, Anastasiya Lukacheva, Georgii Fofanov, Aleksander Kim, Evegeniy Fedorov, Daria Sitovskaya, Alexey Ulitin, Natalia Mikhailova, Ilya Anufriev, Maria Istomina, Ekaterina Murashko, Elizaveta Kessenikh, Nikolay Aksenov, Yulia Vakhitova, Konstantin Samochernykh, Emil Pitkin, Evgeny Shlyakhto, Stephanie E. Combs

**Affiliations:** ^1^ Department of Radiation Oncology, Klinikum rechts der Isar, Technical University of Munich, Munich, Germany; ^2^ Personalized Medicine Centre, Almazov National Medical Research Centre, St. Petersburg, Russia; ^3^ Laboratory of Biomedical Nanotechnologies, Institute of Cytology of the Russian Academy of Sciences (RAS), St. Petersburg, Russia; ^4^ Smorodintsev Research Institute of Influenza, St. Petersburg, Russia; ^5^ Polenov Neurosurgical Institute, Almazov National Medical Research Centre, St. Petersburg, Russia; ^6^ Department of Statistics and Data Science, Wharton School, University of Pennsylvania, Philadelphia, PA, United States

**Keywords:** membrane-bound Hsp70, heat shock protein, multiforme glioblastoma, tumor targeting, diagnostics, fluorescent imaging, fluorescence-guided surgery, intraoperative imaging

## Abstract

Multiforme glioblastoma-homing peptides, particularly targeting plasma membrane-bound heat shock protein mHsp70, demonstrate great application potential for tumor theranostics. In the current study, to further increase the bioavailability as well as penetration capacity through the blood-brain barrier (BBB) of the mHsp70-targeted peptide TKDNNLLGRFELSG, which is known to bind to the oligomerization sequence of mHsp70 chaperone, the latter was conjugated with tripeptide RGD (forming chimeric peptide termed RAS70). In the model BBB system RAS70 efficiently crossed the barrier accumulating in the glioblastoma cells. Subsequently, in the orthotopic glioma models, intravenous administration of the fluorescently labeled agent (RAS70-sCy7.5) resulted in the tumor retention of peptide (further confirmed by histological studies). Thus, as shown by the biodistribution studies employing epifluorescence imaging, accumulation of RAS70-sCy7.5 in C6 glioma was significantly enhanced as compared to scramble peptide. Local application of the RAS70-sCy7.5 peptide that was sprayed over the dissected brain tissues helped to efficiently delineate the tumors in glioma-bearing animals employing an intraoperative fluorescent imaging system. Tumor-specific internalization of the peptide was further confirmed on the *ex vivo* primary GBM samples obtained from adult neurooncological patients. In conclusion, RAS70 peptide demonstrated high glioma-homing properties which could be employed for the intraoperative tumor visualization as well as for developing a potential carrier for drug delivery.

## Introduction

Targeted diagnostics and therapy of malignant tumors, especially glioblastoma multiforme (GBM), which is characterized by rapid infiltrative growth and resistance to chemoradiotherapy, is one of the most important areas in translational and clinical oncology (1). The determining factor in the development of targeted drugs is the choice of a tumor-specific target, which should be present in the majority of the tumor cell population and be accessible to the target drug. Such a promising target may be the 70 kDa heat shock protein HSP70, which is selectively presented on the surface of the plasma membrane of tumor cells, including glioblastoma (2, 3), but not on normal cells (reviewed in (4, 5)). The selectivity of protein expression is due to its electrostatic and hydrophobic interactions with specific lipids of lipid rafts (glycosphingolipid globoyltriaosylceramide Gb3) (6) and other lipids (including phosphatidylserine) (7–9). In a recently published study, the authors showed that primary GBM cells (including cells with stemness properties as shown by SOX2 and Nestin expression) obtained from neuro-oncological patients (adult (n = 23) and pediatric (n = 9)) carry Hsp70 on their surface, which in turn determines highly invasive and migratory activity of the cells (3). mHsp70-positive cells were present both in viable (contrast-accumulating tissue according to the MRI data) tissue and at the tumor border in the perifocal zone (especially in oncostreams). The use of membrane-bound mHsp70 protein as a potential target for theranostics of solid tumors has shown its effectiveness in a number of studies in which the chaperone was targeted by various agents such as monoclonal antibodies (10, 11), peptides (12, 13), anticalins (14), protein granzyme B (15, 16) as well as NK and CAR NK cells (17, 18). Hsp70-targeted molecules were also actively used as nanosized agents in the form of monotherapy or in combination with chemoradiation therapy and immunotherapy (11, 19). Thus, Svirshchevskaya et al. showed that chitosan core-shell nanoparticles (NPs) decorated with anti-HSP70 monoclonal antibodies and containing the model drug allocolchicinoid significantly suppressed the growth of several tumor types including prostate, breast, colon, pancreas cancers and lymphomas (20). In another study by Xie et al., the authors used mHsp70-targeted and acid-triggered self-assembled gold nanocarriers in combination with immunotherapy (PD-1 checkpoint blockade), which resulted in significant slowdown in tumor growth and survival of the glioma-bearing animals (21).

In the current study, a peptide recognizing HSP70 (14-amino acid TKD peptide, TKDNNLLGRFELSG) was chosen as a tumor-targeting agent (12, 13, 22). Peptides due to their lower affinity, shorter half-life (as compared to antibodies) as well as high efficiency in cell internalization and tissues penetration demonstrate a great potential for cancer diagnostics and therapy, including brain tumors (23, 24). Previous studies when mHsp70-recognizing peptide was employed as a PET tracer showed high tumor-specific retention of agent leading to enrichment of tracer in colorectal mice tumor CT26 and 4T1 breast carcinoma, while in mHsp70-negative fibroblastic hyperplasia there was no tracer retention (12). In a more recent study by Holzmann et al. the authors reported fluorescent imaging of human head and neck cancer employing fluorescence labelled peptide TPP-IRDye800 that was sufficient for real-time tissue delineation (25).

To further increase the glioblastoma-homing properties of the Hsp70-targeting peptide as well as its penetration through the blood-brain barrier (BBB) the peptide was conjugated with the tripeptide Arg-Gly-Asp (RGD), which is known to bind to integrins of endothelial cells, particularly αvβ3 and αvβ5 (26). The RGD sequence has been recognized as a minimal integrin sequence present in many natural ligands binding to the αvβ3 receptor, such as fibrinogen, fibronectin, laminin, osteopontin, etc. (27). Currently, RGD is the main motif for many molecules designed to bind to the αvβ3 integrin and other integrins. Due to the conservation of the RGD sequence, the affinity of natural and chemically synthesized ligands is influenced by other amino acid residues flanking the RGD motif. In addition to direct interactions between these residues and the integrin receptor, the flanking groups influence the folding of the peptide and, consequently, the conformational features of the RGD motif. In the study by Bohn et al. it was shown that the labeled RGD peptide [99mTc]-HYNIC-RGD highly and uniformly accumulated in avβ3-positive cell lines compared to avβ3-negative cells and was rapidly cleared from the circulation and excreted via the kidneys (28). A biodistribution analysis of 188Re-RGD peptide after intravenous administration to S180 sarcoma-bearing mice demonstrated the highest tumor uptake of 188Re-HGRGDGRGDF(D) as compared to the short peptide 188Re-HGRGDF(D) (29). The superior tumor targeting performance of 188Re-HGRGDGRGDF(D) might be explained by the 2-fold presence of RGD sequences in this peptide, which provides a higher density of activated binding sites (29). In another study, the biodistribution of the cyclic RGD complex following intravenous administration in U-87MG glioma-bearing mice showed high tumor uptake and low uptake in other non-target tissues except kidney and liver, and intestine for excretion (30). Taking into account the existing data on the glioma-targeting potential of RGD the latter was chosen to conjugate to the mHsp70-targeted peptide in order to increase the tumor-accumulating potential.

Herein, we demonstrate that mHsp70-targeting peptide containing RGD motif (this chimeric peptide was termed RAS70) can efficiently penetrate through the blood-brain barrier and accumulate in the glioblastoma cells. Subsequently, employing orthotopic glioma models in rodents, we confirm the tumor-homing properties of the RAS70 peptide when being either intravenously or locally administered to target the brain tumor.

## Materials and methods

### Synthesis and conjugation of peptides with fluorochromes

The TKDNNLLGRFELSG (TKD), TKD-RGD (TKDNNLLGRFELSG-Beta-Ala-RGD) (termed RAS70) as well as control scramble peptides NGLTLKNDFSRLEG (NGL) and NGLTLKNDFSRLEG-Beta-Ala-RGD (NGL-RGD) were obtained by solid-phase peptide synthesis using Fmoc technology on Wong’s polymer. Fmoc protection was removed using 20% piperidine in dimethylformamide. 1,3-Diisopropylcarbodiimide with the addition of hydroxybenzotriazole was used as a condensing agent. Removal from the resin was carried out using trifluoroacetic acid (TFA) with the addition of scavengers. The peptide was purified by RP-HPLC in a gradient of acetonitrile in 0.1% TFA in water on a chromatographic column Xbridge BEH130 prep C18 OBD 10 μm 130 A 250*19 mm (Waters, USA) to a purity of at least 95%. The purified peptide was lyophilized. Purity analysis was performed using RP-HPLC in a gradient of acetonitrile in 0.1% TFA in water on an analytical chromatographic column Waters Delta-Pak 5 μm 100 A 150*3.9 mm (Waters, USA). The authenticity of the obtained peptide was confirmed by mass spectrometric analysis.

The purified peptides were conjugated with fluorophores in the form of N-hydroxy-succinimide esters: sulfo-Cy5-NHS (CAS 2230212-27-6, cat. No. 63320, Lumiprobe, USA) or sulfo-Cy7,5-NHS (CAS 2736437-44-6, cat. No. 66320, Lumiprobe, USA), Fluorescein 5-carbamoylmethylthiopropansäure-NHS-ester (F9551, Sigma-Aldrich, USA). Conjugation was performed in 0.1 M sodium carbonate buffer pH 8.5 with 10% DMSO for 3 h in the dark, peptides were taken in a 4-fold molar excess relative to the fluorophore. Purification and characterization of conjugates were performed by the same methods and on the same equipment as used for the free peptide. The purified conjugate was lyophilized and stored at –20°C, dissolved immediately before use.

### Evaluation of the RAS70 stability

HPLC grade reagents include acetonitrile (1.00029) and formic acid (5.33002), were purchased from Sigma Aldrich. The deionized HPLC grade water was obtained through a Milli-Q system (Millipore, Milford, MA, USA). Sodium acetate (Sigma Aldrich, St. Louis, USA) was used for MS calibration. Dalargin (Tyrosyl-D-alanyl-glycyl-phenylalanyl-leucyl-arginine; UNII V13505565P) was used as an internal standard. Protein LoBind^®^ Tubes (022431081, Eppendorf, Hamburg, Germany). The stock solution of RAS70 were prepared by dissolving 1 mg of RAS70 reference standard in 1 mL of a sterile solution of 0.9% sodium chloride in water. After centrifugation the supernatant were transferred into Protein LoBind Tubes (Eppendorf, Hamburg, Germany). Working standard solutions for stability study were prepared using a dilution method. All of the solutions were stored away from light at RT and in a refrigerator at +4°C. The samples were analyzed on a HPLC-QTOF/MS system (Bruker Daltonics, Bremen, Germany) that consisted of an Elute UHPLC coupled to Q-TOF Maxis Impact equipped with an electrospray ionization source (**Supplementary Figure S1**). HPLC-separation was performed on a Chromolith Performance RP-18 endcapped 100-2 with Chromolith RP-18e 5-2 mm PreColumn (Merck KGaA, Darmstadt, Germany) at 40°C. The temperature in the autosampler was set at 10°C. The mobile phases consist of 0.1% (v/v) formic acid in water (A) and 0.1% (v/v) formic acid in acetonitrile (B). An aliquot of 5 µL was injected with the following gradient progile: 0.0–3.0 min (10–70%B), 3.0–3.2 min (70–10% B), 3.2–5.0 min (10% B) with the flow rate 0.3 mL/min. The MS-spectra were obtained in the positive mode using a scan range from *m*/*z* 150 to 2200. The acquisition parameters were as follows: Source: end plate offset 500 V, capillary 4500 V, nebulizer 29 psi, dry gas 6.0 L/min, and dry temperature 220 °С. Chromatograms and mass spectra were analyzed using DataAnalysis^®^ 5.0 software (Bruker Daltonics GmbH, Bremen, Germany).

### Cells

The C6 (rat glioma), GL261 (mouse glioma), human T98G, U87, U251 glioblastoma cell lines were provided by the shared research facility “Vertebrate cell culture collection” (Institute of Cytology of the Russian Academy of Sciences, St. Petersburg, Russia). The cells were cultured using DMEM/F-12 medium with the addition of 10% fetal bovine serum (FBS) and gentamicin (Gibco, Carlsbad, USA) at a working concentration of 50 µg/mL at 37°C in a 5% CO_2_. For experiments, a suspension of single cells was obtained using 0.25% trypsin-EDTA (Gibco, Carlsbad, USA).

Primary GBM cells (ANI, IBD, TMS) were isolated from fragments of postoperative material from patients diagnosed with GBM (see (3)). The preoperative diagnosis was confirmed by histological and immunohistochemical assessment of the resected tumors. Biopsy material was provided by the Russian Research Neurosurgical Institute named after Prof. A. L. Polenov (Federal State Budgetary Institution “NMIC named after V. A. Almazov” of the Ministry of Health of the Russian Federation) according to the conclusion of the ethical committee No. 2712-20 dated December 21, 2020. The cells were isolated by mechanical and enzymatic treatment in accordance with the standard protocol (31). The resulting primary GBM cells were cultured at 37°C in a 5% CO_2_ in DMEM/F12 medium (Thermo Fisher Scientific, Waltham, USA) containing HEPES-buffer (Sigma-Aldrich, Burlington, USA), GlutaMAX (Gibco, Carlsbud, USA), B27 without vitamin A (250 µg/mL, Thermo Fisher Scientific, Waltham, USA), EGF and βFGF (20 ng/mL, Gibco, Carsbud, USA) and a mixture of gentamicin (50 µg/mL, Gibco, Carlsbud, USA) and amphotericin B (250 µg/mL, Gibco, Carlsbud, USA). For experiments, a suspension of single cells was obtained using Accutase solution (400 units/ml, Sigma-Aldrich, Waltham, USA).

### MTT-assay

3-(4,5-dimethylthiazol-2-yl)-2,5-diphenyltetrazolium bromide (MTT) assay was performed to evaluate the cytotoxic effect of TKD-sCy5, RAS70-sCy5, and NGL-RGD-sCy5 peptides. The C6, GL261, and T98G cells were seeded in 96-well plates (Thermo Fisher Scientific, Waltham, USA) at concentration of 5*10^3^ cells in 100 µL per well, plates were incubated at 37°C in 5% CO_2_. After 24 hours, peptides were added to the culture medium for final concentration of 7.5, 75, and 750 μg/mL; plates were incubated for 24, 48 and 72 hours. Cells without peptides were used as a control, cells incubated with 1 mg/mL of the cisplatin (CAS-Numb. 15663-27-1, Sigma-Aldrich, USA) – as a positive control. At the end of the incubation period, 1 mg/mL MTT solution was added for 4 hours and dissolved with DMSO. The absorbance was measured at 570 nm using Varioskan LUX Multimode Microplate Reader (Thermo Fisher Scientific, Waltham, USA).

### Confocal microscopy

The binding of the TKD-sCy5, RAS70-sCy5, and NGL-RGD-sCy5 peptides to tumor cells was visualized using the Olympus FV3000 confocal system (Olympus, Japan). The C6, GL261, T98G, ANI, IBD, TMS cells (0.1×10^6^ mL^-1^ cells) were placed on Nunc™ Lab-Tek™ Chamber Slide System (Thermo Fisher Scientific, USA) and kept overnight. For primary GBM cells the chambers were pre-coated with Matrigel (0.2 mg/mL, Corning, New York, USA) to increase adhesion properties. The cells were incubated with peptides at a concentration of 75 μg/mL for 15, 30, and 60 minutes on ice in the dark. After the incubation cells were washed with PBS, fixed in a 10% formalin solution (Sigma-Aldrich, Burlington, USA), and mounted using a mounting medium containing DAPI (Ibidi, Graefelfing, Germany). Unstained cells were used as controls. To assess the mHsp70-mediated uptake of either TKD or RAS70 tumor cells prior to incubation with peptides were pre-treated with anti-Hsp70 monoclonal antibodies SPA-810 (1:250, Stressgen) and SPA-815 (1:250 Stressgen). To evaluate integrin (αvβ3, αvβ5)-mediated penetration of RAS70 tumor cells prior to incubation with peptides were pre-treated with anti- αvβ3 (1:200 clone LM609, ZMS1030, Sigma-Aldrich, USA), -αvβ5 (1:200 clone P1F6, MAB1961Z, Sigma-Aldrich, USA) monoclonal antibodies.

To study the mechanisms of peptide internalization, cells were incubated with peptides for 30 minutes in 37°C in a 5% CO_2_, washed with PBS, fixed in a 10% formalin solution (Sigma-Aldrich, USA), permeabilized with 0,1% Triton-X100 solution (Servicebio, Wuhan, China), blocked with 5% bovine serum albumin solution and incubated overnight with Alexa Fluor^®^ 488 Anti-Rab5 antibody [EPR21801] (#ab270094, abcam, USA), and with Alexa Fluor^®^ 488 Anti-LAMP1 antibody [EPR24395-31] (#281758, abcam, USA). After 24 hours cells were washed with 0,1% Tween-20 solution (Sigma-Aldrich, Burlington, USA) and mounted using a mounting medium containing DAPI (Ibidi, Graefelfing, Germany). To study the co-localization of the peptide with endolysosomal markers the raw confocal images (n = 10 for each group) were obtained in a 3-channel, 1024 × 1024 pixel format. Colocalization coefficient (τ-Kendall rank correlation value) was computed with the Coloc2 plugin and ImageJ 1.54g (32).

To characterize the cells of primary GBM cultures, staining with the following markers was performed. To evaluate the expression of membrane form Hsp70 the cells were stained with Hsp70 Monoclonal antibody (SPA810, StressMarq, Canada). To identify the qualities of stem cells Nestin Monoclonal antibody (10C2), Alexa Fluor 555 (MA1-110-A555, Invitrogen, USA) and SOX2 Monoclonal antibody (Btjce), eFluor 570, eBioscience (41-9811-82, Invitrogen, USA) were used. To identify the expression of neuronal and glial origin markers cells were stained with GFAP Polyclonal antibody (PA5-16291, Invitrogen, USA) and β-3 Tubulin Monoclonal antibody (2G10) (MA1-118, Invitrogen, USA). To evaluate the expression the proliferative properties cells were stained with Ki-67 Polyclonal antibody (PA5-16446, Invitrogen, USA).

### Transwell analysis

To assess the penetrating properties of the peptides, a transwell system was used, where human umbilical vein endothelial cells (HUVEC) (5 × 10^5^ cells) and astrocytes (5 × 10^5^ cells) were seeded at the bottom of the upper chamber, and tumor cells (C6, GL261, U87, U251) (1 × 10^4^ cells) were seeded at the bottom of the lower chamber (33). Then, a peptide labeled with a fluorescent label was introduced into the upper chamber and co-incubation was carried out for 12, 24 and 36 hours. At the end of the incubation, the tumor cells were washed with cold phosphate-buffered saline (PBS) and fixed with a 4% solution of paraformaldehyde in PBS. Nuclei were stained with 4’,6-diamidino-2-phenylindole (DAPI). Fluorescent images were obtained using a Leica TCS SP8 confocal microscope (Leica, Germany). To avoid possible cross-interference of different fluorochromes, images for DAPI and FITC were obtained using a sequential sample scanning method. Fluorescently labeled cells were counted in the lower chamber. To estimate the mHsp70-mediated uptake of chaperone-targeted peptides tumor cells prior to incubation were pre-treated with anti-Hsp70 monoclonal antibodies SPA-810 (1:250, Stressgen) and SPA-815 (1:250 Stressgen). To exclude integrin (αvβ3, αvβ5)-mediated penetration of RAS70 tumor cells prior to incubation with peptides were pre-treated with anti- αvβ3 (1:200 clone LM609, ZMS1030, Sigma-Aldrich, USA), -αvβ5 (1:200 clone P1F6, MAB1961Z, Sigma-Aldrich, USA) monoclonal antibodies.

### Сell motility analysis

The cell speed of the primary GBM culture was determined using Image ExFluorer, an automatic cell imaging device (LCI, Korea). The cells were seeded into 24-well plates, and the cell nuclei were labelled with the fluorescent dye Hoechst 33342. The plates were then placed in an integrated incubator with temperature control (37°C) and 5% CO_2_. The fluorescence was stimulated at 405 nm using a long-focus semi-apochromatic 20× lens with a numerical aperture of 0.45. The primary IBD cell line was divided into 4 wells, each with five visual fields: two untreated wells on distinct matrices (Collagen I and Fibrinogene) and two wells containing RAS70 peptide on matrices.

FOVs were captured every 15 minutes over 56 hours, resulting in images with a resolution of 2.560 × 2.160 pixels (0.33 μm/px). Real-time focus correction was used. The captured frames were analyzed using the NIS-Elements software with a module for automatic segmentation, quantification, and tracking of individual cells. Cell nuclei (with a diameter of more than 2.5 μm) were recognized in images, and the X-Y coordinates of the nuclei were recorded. Cell movement tracks were built according to the coordinates of the nuclei, and the motility metrics (average speed, maximal speed, and track straightness) were calculated.

### FACS analysis

The binding of the TKD-sCy5, RAS70-sCy5, and NGL-RGD-sCy5 peptides were analyzed using a CytoFLEX flow cytometer (Beckman Coulter, USA). A single cell suspension of C6, GL261, T98G, U87, U251, ANI, IBD, TMS (0.15×10^6^ cells in mL^-1^) were incubated for 30 minutes on ice in the dark with peptides in concentration of 75 μg/mL, and then washed with cold PBS solution. Cells without peptides were used as controls. To evaluate incorporation of the fluorescently-labeled peptides for longer period of co-incubation of 24 h cells (C6, GL261, U87, U251) were treated with FITC-labeled peptides, then washed with PBS and assessed on a CytoFLEX flow cytometer (Beckman Coulter, USA). Following FACS analysis cell pellet was further evaluated employing confocal microscopy Leica TCS SP8 (Leica Microsystems, Germany).

To identify the surface markers of primary glioblastoma cells, mHsp70 and CD133 staining was performed using Hsp70 monoclonal antibody (SPA810, StressMarq, Canada) and CD133 Monoclonal Antibody, PE (ab252128, Abcam, USA), respectively.

### Animals

Male Wistar rats (The Animal Breeding Facility, Branch of Shemyakin & Ovchinnikov Institute of Bioorganic Chemistry of the Russian Academy of Sciences), with body weights ranging 250 – 300 g, were housed in standard rat cages (480 × 270 × 200 mm) in groups of 2 animals. Male C57BL/6 mice (The Animal Breeding Facility, Branch of Shemyakin & Ovchinnikov Institute of Bioorganic Chemistry of the Russian Academy of Sciences), with body weights 25 g (6–8 weeks), were housed in standard mouse cage (400 х 200 х 160 mm) in groups of 3 animals. For all animals the following conditions were preserved - circadian 12h light/dark regime; temperature: 22–23°C and humidity: 40–45%. Food and water were at disposal *ad libitum*. After the experiment the animals were euthanized by overdose of anesthetics. All of the procedures used in this study were approved by animal welfare committee of the Almazov National Medical Research Centre on Research Animal Care (protocol PZ_23_11_2_ShevtsovMA_V3), in accordance with the Public Health Service Guide for the Care and Use of Laboratory Animals.

### Orthotopic brain tumors

The animals were anesthetized with a combination of Zoletil 100 (Virbac, France) and Rometar (Bioveta, Czech Republic). Rat glioma C6 (2х10^6^) and mice glioma GL261 (1х10^6^) cells were injected intracranially into the area of *nucl. caudatus dexter*. After 3–4 weeks, the animals demonstrated symptoms of developing a brain tumor.

### Intravenous and topical administration of RAS70

Fluorescence analysis *in vivo* and *ex vivo* was performed on a noninvasive highly sensitive IVIS Lumina LT series III bioimager (PerkinElmer, USA). The study was performed on mice of the C57BL/6 line and Wistar rats (Pushchino Laboratory Animal Nursery). This study was conducted in strict accordance with the recommendations of the Guide for the Care and Use of Laboratory Animals of the National Institutes of Health. The protocol was approved by the Institutional Animal Care and Use Committee (IACUC) of the Almazov National Medical Research Center (approval number: No. AP_23_11_2_Shevtsov MA_V3).

#### Intravenous delivery of tumor-targeted peptides

For evaluation of the tumor retention of the RAS70 peptide in GL261 glioma-bearing mice the animals following tumor cells injection were randomly assigned to the study groups (6 animals each) as follows (1): control group – PBS i.v. injection via tail vein (100 μL) (2); i.v. administration of NGL-sCy7.5 peptide (100 μl, 2.5 mg/kg) (3); i.v. administration of NGL-RGD-sCy7.5 peptide (100 μl, 2.5 mg/kg) (4); i.v. administration of TKD-sCy7.5 peptide (100 μl, 2.5 mg/kg) (5); i.v. administration of NGL-sCy7.5 peptide (100 μl, 2.5 mg/kg). Following clinical manifestation of the brain tumor progression in animals (3–4 weeks after intracranial tumor cells injection) peptides were i.v. injected and the peptides biodistribution was assessed employing IVIS *in vivo* Imaging System (PerkinElmer, USA).

For assessment of the tumor accumulation of the RAS70 peptide in C6 glioma-bearing Wistar rats the animals following tumor cells injection were randomly assigned to the study groups (6 animals each) as follows (1): control group – PBS i.v. injection via tail vein (200 μL) (2); i.v. administration of NGL-sCy7.5 peptide (200 μl, 2.5 mg/kg) (3); i.v. administration of NGL-RGD-sCy7.5 peptide (200 μl, 2.5 mg/kg) (4); i.v. administration of TKD-sCy7.5 peptide (200 μl, 2.5 mg/kg) (5); i.v. administration of NGL-sCy7.5 peptide (200 μl, 2.5 mg/kg). Following clinical manifestation of the brain tumor progression in animals (3–4 weeks after intracranial tumor cells injection) peptides were i.v. injected and animals the biodistribution was assessed employing Bioimager.

To non-invasively monitor the biodistribution and accumulation of TKD, NGL, NGL-RGD, and RAS70 peptides in tissues after intravenous administration, rodents were pre-anesthetized with 2% isoflurane-based anesthesia (“AERRAN”, Baxter) in a pre-training box. The animals were then placed in a chamber where they were individually anesthetized with 1% masked anesthesia using an integrated anesthesia line throughout the imaging period. The body temperature of the animals was maintained at 37°C during imaging by a built-in thermostat. Fluorescence detection was performed at the following time points: 0, 24, and 48 h after intravenous peptide administration. Peptides were pre-cross-linked with the fluorescent dye sCy7.5 to enable detection. The imaging parameters of the Bioimager were adjusted to the dye, corresponding to wavelengths of 650 nm for absorbance and 785 nm for emission. Visualization was performed with the following instrument settings: binning - 4, exposure - (1 - 30 s auto). After the biodistribution study, the accumulation of peptides in organs and tissues *ex vivo* was evaluated.

Processing of the obtained luminescence images was performed using Living Image 4.7.4 firmware. The luminescence signals were read from the color maps of the obtained images by selecting the ROI contour (region of interest). ROIs covering accumulation areas and organs were manually delineated, after which the program calculated the total radiant efficiency for each ROI. Thus, the total radiation efficiency is proportional to both the surface area of the organ in the image (ROI area) and the fluorescence signal of each pixel in the ROI.

The *ex vivo* study was performed on mouse organs and tissues after intravenous administration of peptides at 24 and 48 hours and after local application of peptides in models of intracranial brain glioma. The organs of interest such as brain, tumor, heart, lung, kidney, spleen, liver, and small intestine were analyzed using identical fluorescence imager settings as for the *in vivo* study, and the ROI area was highlighted along the contour of the organs to assess the luminescence intensity.

#### Topical application

To assess the local accumulation of the fluorescently-labeled peptides the latter were sprayed over the freshly dissected brain tissues from either C6 glioma-bearing rats or GL261 glioma bearing mice. For this animals following tumor cells injection were randomly assigned to the study groups (6 animals each) as follows (1): control group – PBS (50 μL) (2); NGL-sCy7.5 peptide (50 μl, at concentration of 100 μg) (3); NGL-RGD-sCy7.5 peptide (50 μl, at concentration of 100 μg) (4); TKD-sCy7.5 peptide (50 μl, at concentration of 100 μg) (5); NGL-sCy7.5 peptide (50 μl, at concentration of 100 μg). The whole sample was sprayed with peptide and the exposure time constituted 5 minutes at room temperature. Following rinsing of the brain tissues with PBS solution the images were obtained employing IVIS Lumina LT series III bioimager (PerkinElmer, USA).

### Patients

To study RAS70 peptide accumulation in the brain tumors the latter were obtained from neuro-oncological patients. Intraoperative biopsy material obtained from adult (n=6) and pediatric (n=2) patients with radiological diagnosis of high-grade gliomas without previous radiation therapy and chemotherapy. The patients underwent microsurgical interventions between February 2024 and July 2024 (**Table 1**). The study protocol was approved by the Ethics Committee of the Almazov Medical Research Centre (approval No. 2712-20, December 21, 2020).

**Table 1:** Patients and tumor characteristics.

Patient code	Age (years)	Sex	KPS before surgery	Dex. Before surgery (mg)	Tumor site (lobe)	Hemisphere	Histology	MIB-1 index (%)	EGFR	ATRX	MGMT
BAV	57	male	70	4	parietal	left	GB IDH1-wt	12	++	Loss of expression in 70% of cells	:
IEI	77	male	70	8	occipital	right	GB IDH1-wt	35	*	Loss of expression in 70% of cells	:
NYM	56	male	70	4	parietal	left	GB IDH1-wt	25	*	Loss of expression in 10% of cells	*
FSK	36	female	70	8	frontal	left	Anaplastic astrocytoma IDH1-mutant	15	++	Subtotal loss of expression	*
ZLS	80	female	60	12	temporal	left	GB IDH1-wt	20	*	Loss of expression in 5% of cells	*
RGN	75	female	60	16	parietal	right	GB IDH1-wt	20	:	Subtotal loss of expression	*
IBD	11	male	70	2	thalamus	left	Paediatric-type diffuse HGG IDH1-wt	:	:	*	:
TMS	3	female	70	:	temporal	left	Paediatric-type diffuse HGG H3-wt, IDH1-wt	:	:	*	:

KPS, Karnofsky Performance Scale; Dex, Dexamethasone; GB, glioblastoma; HGG, high-grade glioma; wt, wildtype; * - not evaluated, MGMT «-» negative nuclear staining, «++» - positive nuclear staining in 10-50% of tumor cells; EGFR «-» negative cytoplasmic staining, «++» - positive cytoplasmic staining in <50% of tumor cells.

All patients gave their informed consent for inclusion before they participated in the study; all experiments were performed in accordance with relevant named guidelines and regulations.

The tumor sample was obtained intraoperatively from the defined contrast-enhancing region designated on the MR image. Preoperative MRI scanning of the brain was performed on an expert-class tomograph (MAGNETOM Skyra 3,0T, Siemens, Germany) applying a standard imaging protocol (34). MRI sequences included axial T1 FSE (TSE) (slice thickness < 5 mm TR 400–650 ms, TE 10–20 ms), axial T2 FLAIR FSE (TSE) (slice thickness < 5 mm TR 8000–12000 ms, TE 95–130 ms, TI 2200–2850 ms), axial DWI (EPI) (slice thickness – 5 mm, b value – 500, 1000), axial sagittal, coronal postcontrast T1 FSE (TSE) +3D T1 (slice thickness – 1mm). The study included patients with a radiological diagnosis of high-grade glioma: tumors with a distinct ring-like pattern of contrast enhancement with thick irregular walls on MRI and a core area of reduced signal suggestive for tumor necrosis. Patients with tumors located in midline, basal ganglia, or brain-stem tumors were excluded.

Surgical treatment of patients was carried out in accordance with the Clinical Guidelines of the Association of Neurosurgeons of Russia (2020). High-grade glioma samples were obtained from three spatially distinct tumor regions based on preoperative MRI scanning (as was previously proposed by (35). These regions were: necrotic zone - tumor сenter, hypointense on T1 and non-enhancing; the contrast enhanced tumor zone; peritumoral region - ≥ 3mm from enhancing margin and also within hyperintense FLAIR. Histopathological tumor typing and grading were based on the standard criteria of the 5^th^ edition of the World Health Organization (WHO) classification of central nervous system (CNS) tumors (2021) (36). In the pathology department, the material was fixed in 10% buffered formalin (pH = 7.0), dehydrated in a standard manner and embedded in paraffin. Histological sections stained with hematoxylin and eosin were studied, as well as the results of immunohistochemical (IHC) reactions with the following antibodies: anti-IDH1R132H (Dianova, Germany, Dia-H09), 1:300; anti-ATRX (Abcam, USA, ab188029), 1:300; anti-EGFR (Abcam, USA, ab52894) 1:100, anti-MGMT (NovusBiologycals, USA, NB100-168), 1:300; anti-Ki67 (Dako, USA, M7240), 1:50. The EnVision polymer detection system (Dako, CA, USA) was also used. For visualization, the streptavidin–peroxidase polymer ultrasensitive system and DAB chromogen (Sigma-Aldrich, Darmstadt, Germany) were used. The sections were counterstained with Gill’s hematoxylin and then embedded in Bio Mount HM synthetic embedding medium (BIO-OPTICA, Milano, Italy). Additionally, reactions lacking primary antibodies were carried out to ensure the specificity of the observed staining. Histological analysis and microphotography were performed using a scanning microscope Leica Aperio AT2 and the image manager AperioImageScope (Leica Microsystems, USA). Quantification of the results of IHC reactions was performed by counting positively stained cells in sections of the brain tissue (х400 in 1 mm 2 slices, ImageG).

For evaluation of targeting properties of RAS70 peptide in the non-tumorous brain tissues patient-derived samples obtained intraoperatively from patients with drug-resistant epilepsy underwent temporal resection (n=2) were employed.

#### Topical application of RAS70 in brain tumor *sp*ecimen

To evaluate local accumulation of the fluorescently-labeled peptides the latter were sprayed over the freshly dissected brain tumor specimens as follows (1): control group – PBS (50 μL) (2); NGL-sCy7.5 peptide (50 μl, at concentration of 100 μg) (3); NGL-RGD-sCy7.5 peptide (50 μl, at concentration of 100 μg) (4); TKD-sCy7.5 peptide (50 μl, at concentration of 100 μg) (5); NGL-sCy7.5 peptide (50 μl, at concentration of 100 μg). The whole sample was sprayed with peptide and the exposure time constituted 5 minutes at room temperature. Following rinsing of the brain tissues with PBS solution the images were obtained employing Leica TCS SP8 (Leica Microsystems, Germany) inverted microscope.

### Confocal microscopy of tumor samples

Small pieces of brain tissue (50-100 mm³) were taken from patients or animals during surgery. The resulting material was washed three times in a PBS solution at room temperature and then stained, gently shaking, for 1 hour with one of the following combinations of dyes: a mixture of Hoechst 33342 (0.1 μg/mL) [Invitrogen, USA], monoclonal antibodies against Hsp70 conjugated with FITC, and RAS70 conjugated with Cy5; a mixture of Hoechst 33342 (0.1 μg/mL), LumiTracker^®^ Mito Green FM [Lumiprobe, Russia], and TKD/NGL-RGD/RAS70 conjugated with sCy5. Following staining, the material was rinsed with PBS and put in a thin-bottomed Ibidi μ-plate 35-mm dish. The sample was then covered with a cover glass to achieve equal adherence to the dish’s bottom surface. A Leica TCS SP8 (Leica Microsystems, Germany) inverted microscope fitted with argon and helium-neon lasers was then used to perform confocal laser scanning microscopy on the material. Hoechst 33342 fluorescence was excited with a 405 nm laser and recorded in the range 410 to 550 nm; Hsp70-FITC and Mito Green fluorescence were excited with a 488 nm laser and recorded in the range 495 to 550 nm; and Cy5 fluorescence was excited with a 633 nm laser and recorded in the range 635 to 750 nm. An oil immersion lens, HC PL APO 63x/1,40 OIL CS2, was employed. Three channels were scanned separately, with a confocal aperture width of 100 μm. Images were captured at a resolution of 1024 by 1024 pixels, with an average of three per scanning line. All of the images were taken using the same laser power and detector voltage settings throughout the experiment.

### Statistics

When evaluating group means of two continuous normally distributed variables the parametric Student test was employed. The Man-Whitney test was used for non-normally distributed variables. The significance level for all tests was alpha = 0.05. All confidence intervals were reported at the 95% level. When performing the comparison of multiple groups as a nonparametric analog to the one-way ANOVA test we employed Kruskal-Wallis test. Statistica Version 9.2 for Windows or the R programming language was used for all tests.

When analyzing the results of single-cell velocity measurements, the data for each group (n=25) where first tested for compliance with normal distribution using the Shapiro-Wilk test. In all measurement groups, the data were not normally distributed, so the nonparametric Mann-Whitney test was used to identify differences from the control group. The differences were considered statistically significant at P < 0.05. GraphPad Prism version 9.0.1. was utilized for statistical testing. The colocalization analysis used the Mann-Whitney U-test with Dunnett corrections for multiple comparisons to compare the measurement data from each experimental point (TKD, RAS70) with the NGL-RGD group of images. Statistical significance was defined as P-values below 0.05.

## Results

### RAS70 stability study

The stability of the RAS70 peptide solution was estimated by the area of the chromatographic peak of RAS70, normalized to the peak area of the internal standard, in comparison with freshly prepared working solutions. The observed fluctuations in the area of the chromatographic peak of RAS70 were associated with the rapid sorption of this peptide on both plastic and glass chromatographic vials and inserts. The purity and yield of the synthetic peptide product constituted ≥90%. It was found that the RAS70 solutions were stable for 1 week for both storage conditions (at +4°C and RT) (**Supplementary Figure S2**).

### RAS70 peptide targets mHsp70-positive brain tumor cells

**Figure 1:**
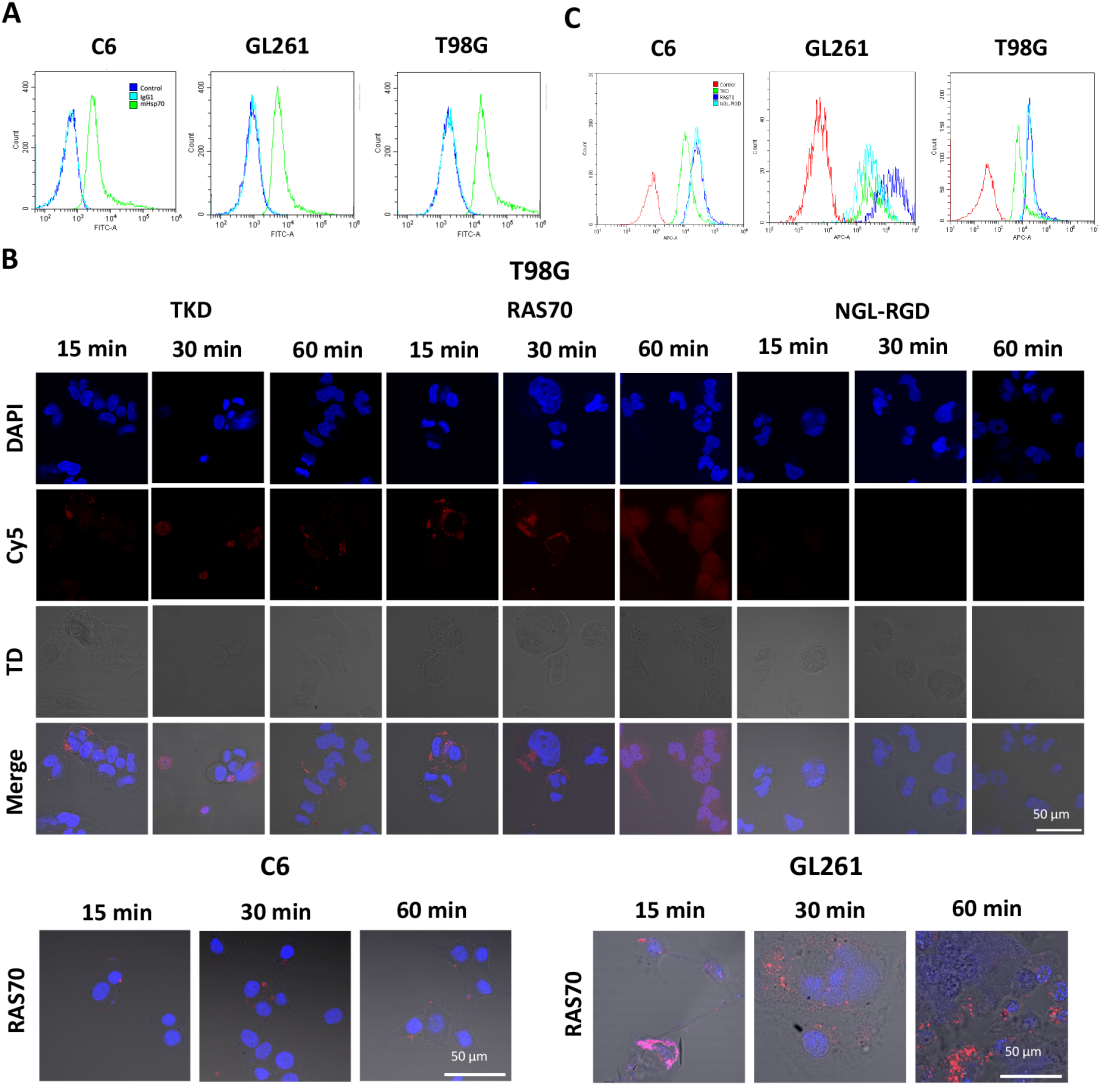
RAS70 peptide specifically accumulates in mHsp70-positive glioblastoma cells. **(A)** Flow cytometry of mHsp70-positive C6, GL261, and T98G cell lines. **(B)** Confocal microscopy images of co-incubation of the TKD, RAS70, and NGL-RGD peptides with tumor cells for 15, 30 and 60 minutes on ice at +4°C. Nuclei were detected with DAPI; peptides were labeled with fluorescent dye Cy5; TD is transmitted light detector. Scale bars, 50 μm. **(C)** The flow cytometry histograms of tumor cells stained with TKD, RAS70, and NGL-RGD peptides.

To study the target properties of the RAS70 peptide, C6 rat glioma, GL261 mice glioma and human T98G glioblastoma cell lines were selected that highly express the Hsp70 protein on their plasma membrane (according to flow cytometry and confocal microscopy data) (**Figure 1A**). Thus, about 100% of C6, GL261, and T98G cells are mHsp70-positive.

**Figure 2:**
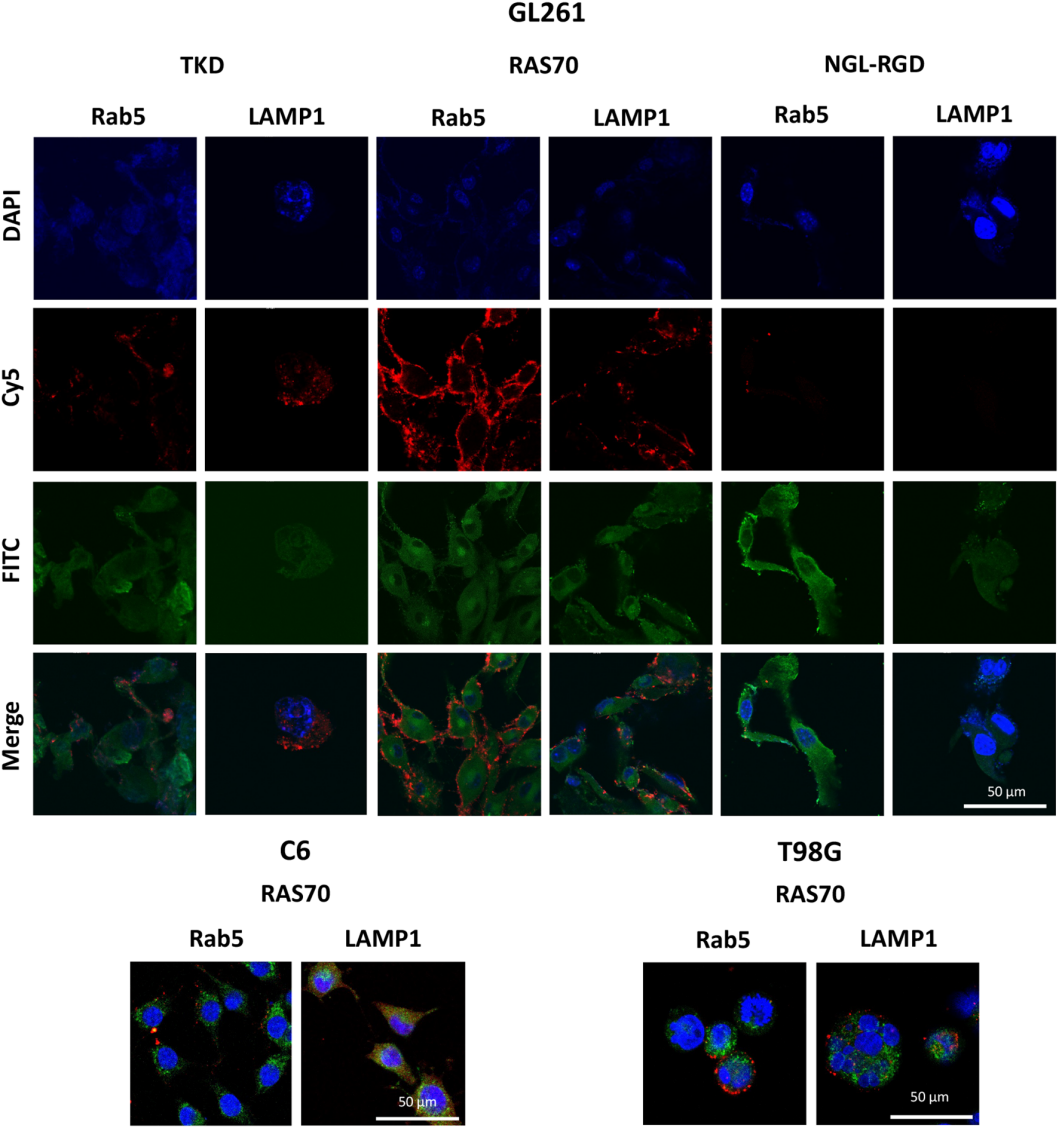
RAS70 peptide penetrated mHsp70-positive glioblastoma cells via endolysosomal pathway. Confocal microscopy images of peptides co-localization with markers of endolysosomes. Nuclei were detected with DAPI; peptides were labeled with fluorescent dye Cy5; early endosomes and lysosomes were detected with FITC-labeled antibodies against Rab5 and LAMP1, respectively. Scale bars, 50 μm.

**Figure 3:**
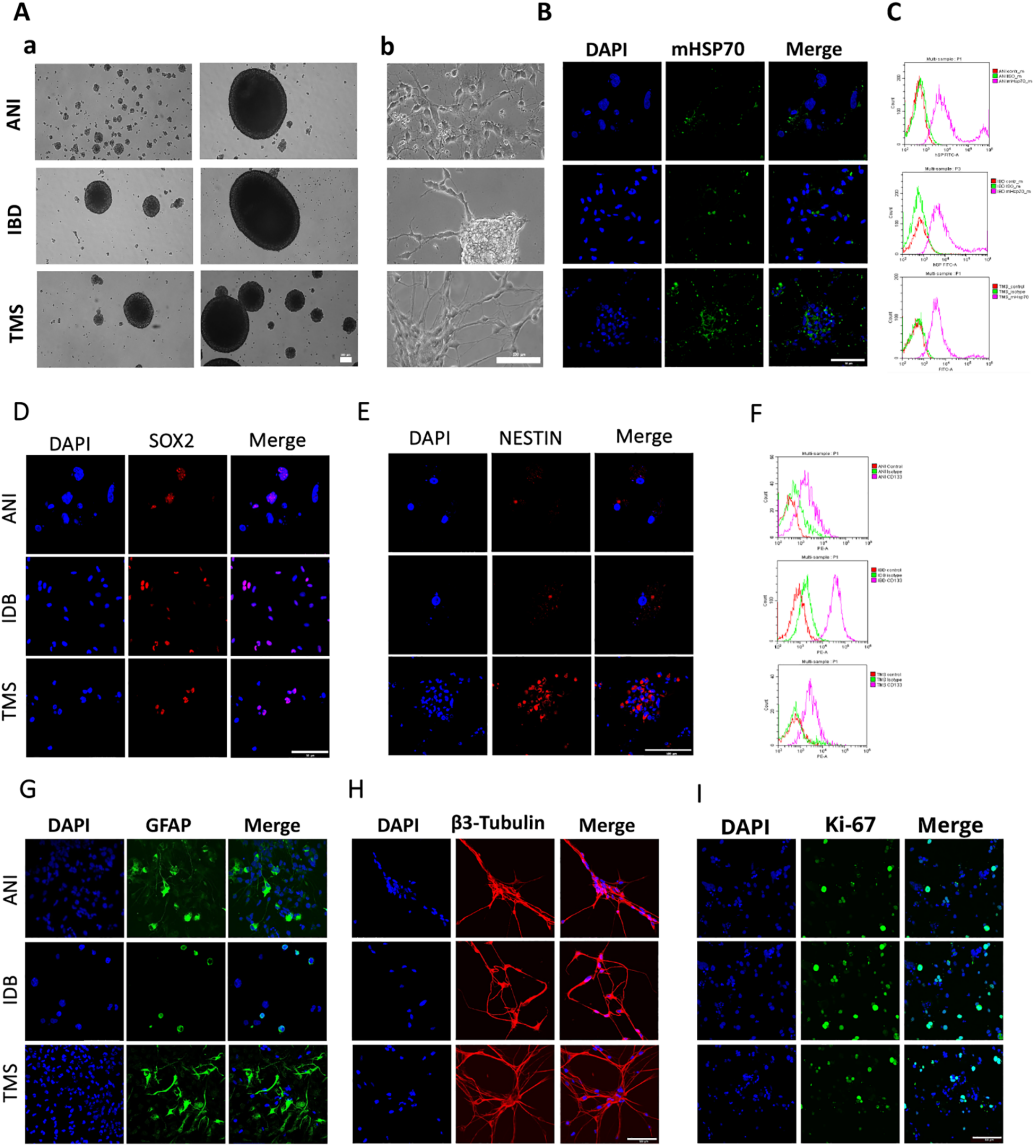
The characteristics of the primary human GBM cell cultures. **(A)** The morphology of the primary human GBM cells – a) the formation of spheroids, b) the adhesive state of cells. **(B, D, E, G–I)** Representative confocal microscopy images of the primary human GBM cells stained for mHsp70 (green), SOX2 (red), NESTIN (red), GFAP (green), β3-Tubulin (red), Ki-67 (green), DAPI (blue). Scale bars, 50 and 100 µm. **(C, F)** Expression of mHsp70 and CD133 measured by flow cytometry.

**Figure 4:**
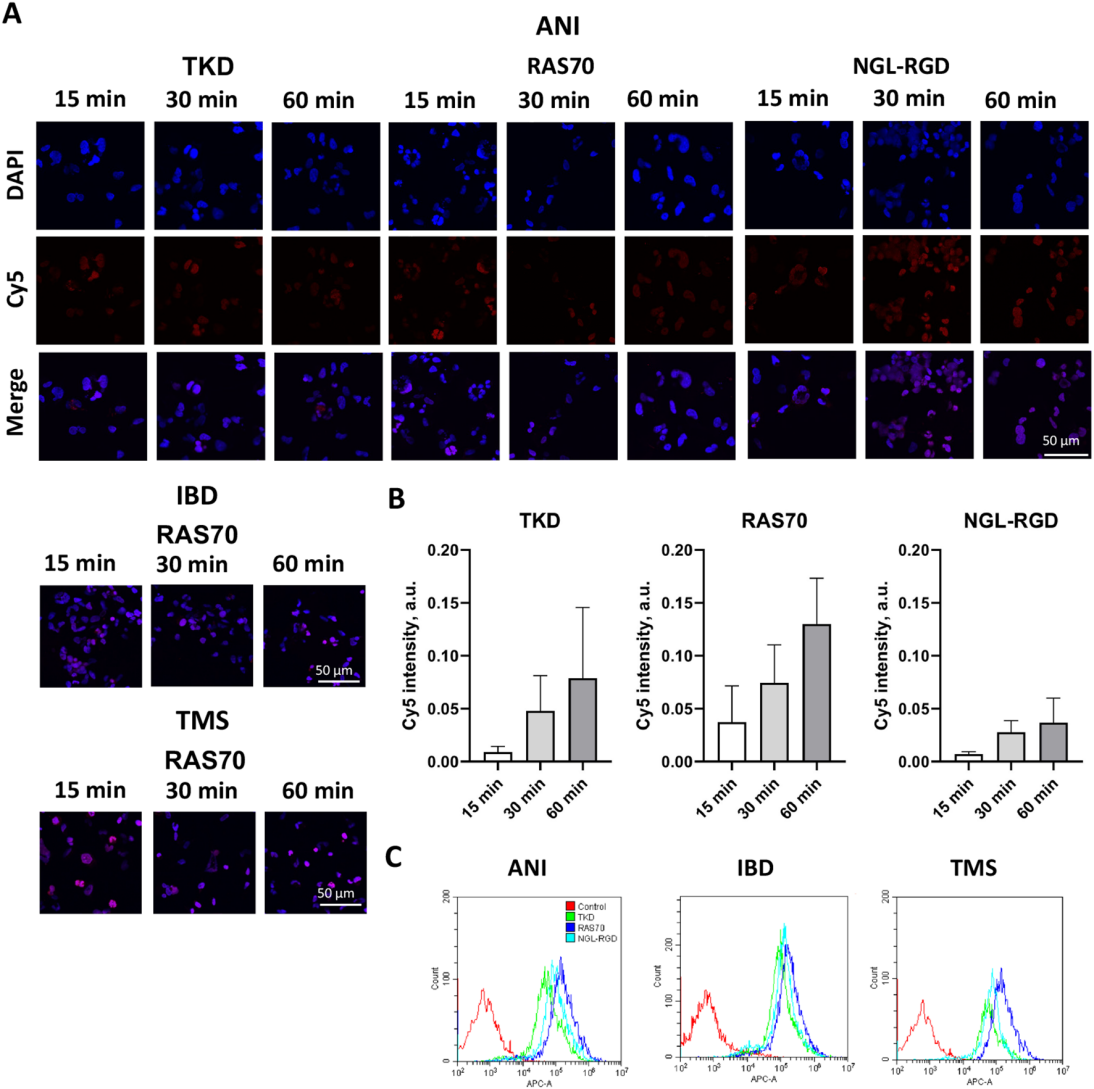
RAS70 peptide targets primary glioblastoma cells. **(A)** Confocal microscopy images of co-incubation of the TKD, RAS70, and NGL-RGD peptides with tumor cells for 15, 30 and 60 minutes at +37°C. Nuclei were detected with DAPI; peptides were labeled with fluorescent dye Cy5. Scale bars, 50 μm. **(B)** Evaluation of the red channel pixel intensities in confocal microscopy images of ANI cells. **(C)** The flow cytometry histograms of tumor cells stained with TKD, RAS70, and NGL-RGD peptides.

**Figure 5:**
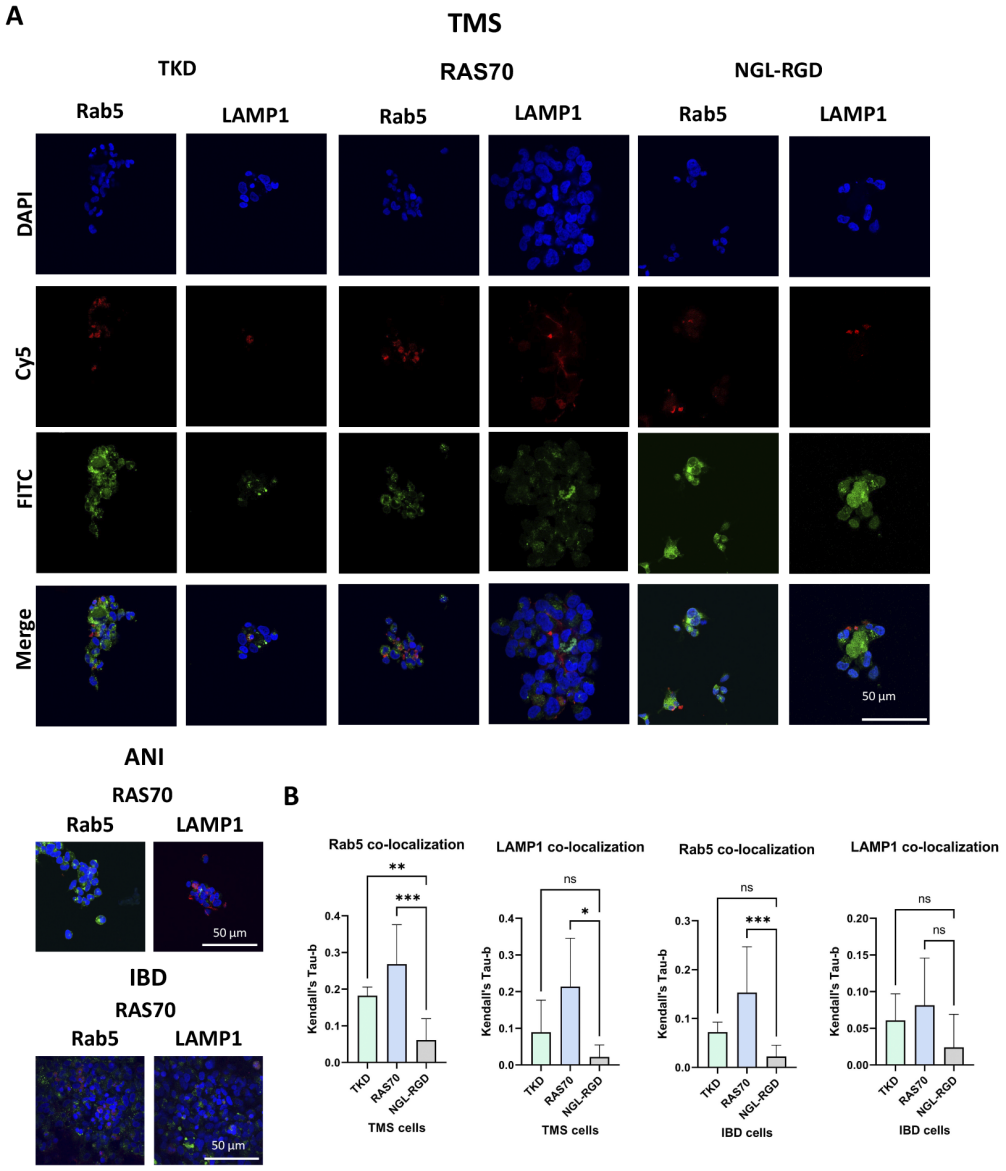
RAS70 peptide accumulates in primary glioblastoma cells via endolysosomal pathway. **(A)** Confocal microscopy images of peptides co-localization with markers of endolysosomal pathway. Nuclei were detected with DAPI; peptides were labeled with fluorescent dye Cy5; early endosomes and lysosomes were detected with FITC-labeled antibodies against Rab5 and LAMP1, respectively. Scale bars, 50 μm. **(B)** Evaluation of the colocalization of proteins and peptides in cells from various patients. Significant differences identified by the Mann-Whitney U-test with Dunnett corrections for multiple comparisons are shown as asterisks (*p < 0.05, **p < 0.01, ***p < 0.001). ns, non-significant.

The next step was co-incubation of the target peptide RAS70 labeled with fluorescent dye Cy5 with tumor cells for 15, 30 and 60 minutes, followed by confocal microscopy analysis. Already after 15 minutes, internalization of the TKD peptide by tumor cells was noted, reaching maximum absorption of the peptide after 60 minutes (**Figures 1B, C**). When RAS70 peptide was applied a higher accumulation of the peptide was detected as compared to TKD or control (i.e., NGL, NGL-RGD) peptides. The peptide RAS70 was predominantly localized in the cytoplasm of cells without penetrating into the nuclei. Subsequent analysis for co-localization of the peptide with markers of early endosomes (Rab5) and lysosomes (LAMP-1) showed that the peptide only partially penetrated the cell via the endolysosomal pathway (**Figure 2**). To exclude processes of active internalization of the peptide, co-incubation of the cells on ice at +4°C was performed. In this case, a significant decrease in the penetration of the RAS70 peptide into the cells was observed (*data not shown*). Next, we evaluated the targeting properties of RAS70 on primary glioblastoma cells (ANI, IBD, TMS) obtained from neuro-oncological patients (3) that were additionally characterized for mHsp70, SOX2, NESTIN, GFAP, β3-Tubulin, Ki-67, and CD133 (**Figures 3A–I**). These GBM cells also exhibited the stemness properties (as shown by SOX2, Nestin co-expression). Already after 15 minutes of co-incubation with RAS70-sCy5 peptide we observed the retention of the peptide in GBM cells (**Figures 4A–C**) with a maximum of accumulation at 60 min partially via the endolysosomal pathway (**Figures 5A, B**).

**Figure 6:**
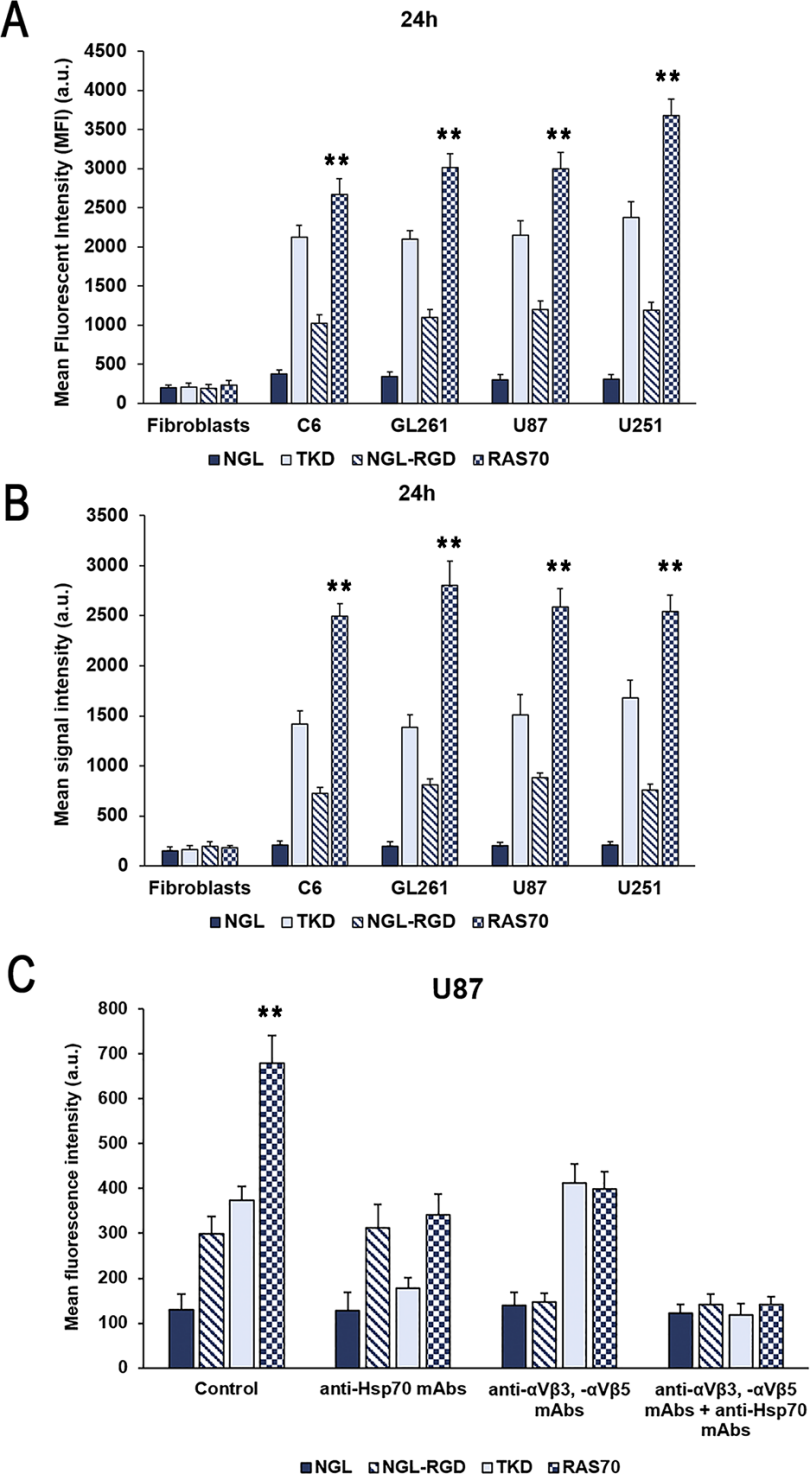
Evaluation of the mHsp70-targeting peptides internalization by glioma cells. **(A)** Flow cytometric evaluation of FITC-labeled peptide accumulation in tumor cell lines (rat glioma C6, mouse glioma GL261, human glioblastoma U87 and U251) after co-incubation for 24 hours. Dermal fibroblasts that do not express membrane-bound mHsp70 protein were used as a control. Data from 3 independent experiments are presented as M ± SE. **(B)** Confocal microscopy assessment of FITC-labeled peptide accumulation in tumor cell lines (rat glioma C6, mouse glioma GL261, human glioblastoma U87 and U251) after co-incubation for 24 hours. Data from 3 independent experiments are presented as M ± SE. **(C)** Analysis of the peptides uptake by human U87 glioblastoma cells following co-incubation for 24 hours. Additionally, prior to incubation with peptides cells were pre-treated with anti-Hsp70 mAbs, anti- αvβ3 and -αvβ5 monoclonal antibodies or by combination of both. Data from 3 independent experiments are presented as M ± SE. ** p < 0.01 for testing mean expression levels.

The incorporation of peptides over longer periods of co-incubation with brain tumor cells was additionally analyzed using a Cytomics FC500 flow cytometer (BeckmanCoulter, USA). Tumor cells (C6, GL261, U87, U251) were incubated with peptides (concentration 50 μg/mL) for 24 hours, washed with PBS. Internalization of FITC-labeled peptides was assessed by determining the mean fluorescence intensity (MFI). According to the study data (**Figure 6A**), it was shown that TKD peptide accumulated in all cell lines – 2120 ± 152 (C6), 2099 ± 107 (GL261), 2145 ± 189 (U87), 2373 ± 201 (U251) compared to the control scramble peptide (NGL) – 378 ± 48 (C6), 344 ± 57 (GL261), 301 ± 66 (U87), 313 ± 56 (U251) (P<0.001). The use of the RAS70 peptide resulted in a more significant accumulation of the agent in the cellular cytoplasm – 2671 ± 201 (C6), 3012 ± 182 (GL261), 2999 ± 209 (U87), 3678 ± 212 (U251) (P<0.001). In mHsp70-negative dermal fibroblasts, which were used as a negative control, we did not detect the accumulation of the targeted peptides TKD and RAS70. Following FACS analysis the cell pellet was collected and evaluated for peptides cell uptake employing confocal microscopy (**Figure 6B**). The obtained microscopy data confirmed higher accumulation of RAS70 peptide in studied cell lines as compared to TKD or scramble peptides.

To evaluate the mHsp70-mediated uptake of the peptides prior to co-incubation with either TKD or RAS70 peptides cells were pre-treated with anti-Hsp70 antibodies (SPA-810, SPA-815) that are known to target the membrane-bound chaperone on cancer cells (37). After application of anti-Hsp70 mAbs we observed a significant decrease of the TKD and RAS70 internalization by cancer cells (but not of control scramble peptides NGL and NGL-RGD) (**Figure 6C**). The tripeptide RGD (Arg-Gly-Asp) motif is known to bind to integrins, including αvβ3 and αvβ5 expressed on brain tumor cells and tumor endothelial cells (38, 39). In order to prove the role of these receptors in RAS70 uptake we employed anti- αvβ3 and -αvβ5 monoclonal antibodies prior to incubation with peptides. As expected, we observed the decrease of NGL-RGD and RAS70 internalization by tumor cells but not of TKD or NGL peptides (**Figure 6C**). When both, anti-Hsp70 and anti-αvβ3/-αvβ5 mAbs, were used we detected a significant abrogation of TKD and RAS70 uptake (as well as of NGL-RGD but not the NGL peptide).

### Analysis of the peptides cytotoxicity

**Figure 7:**
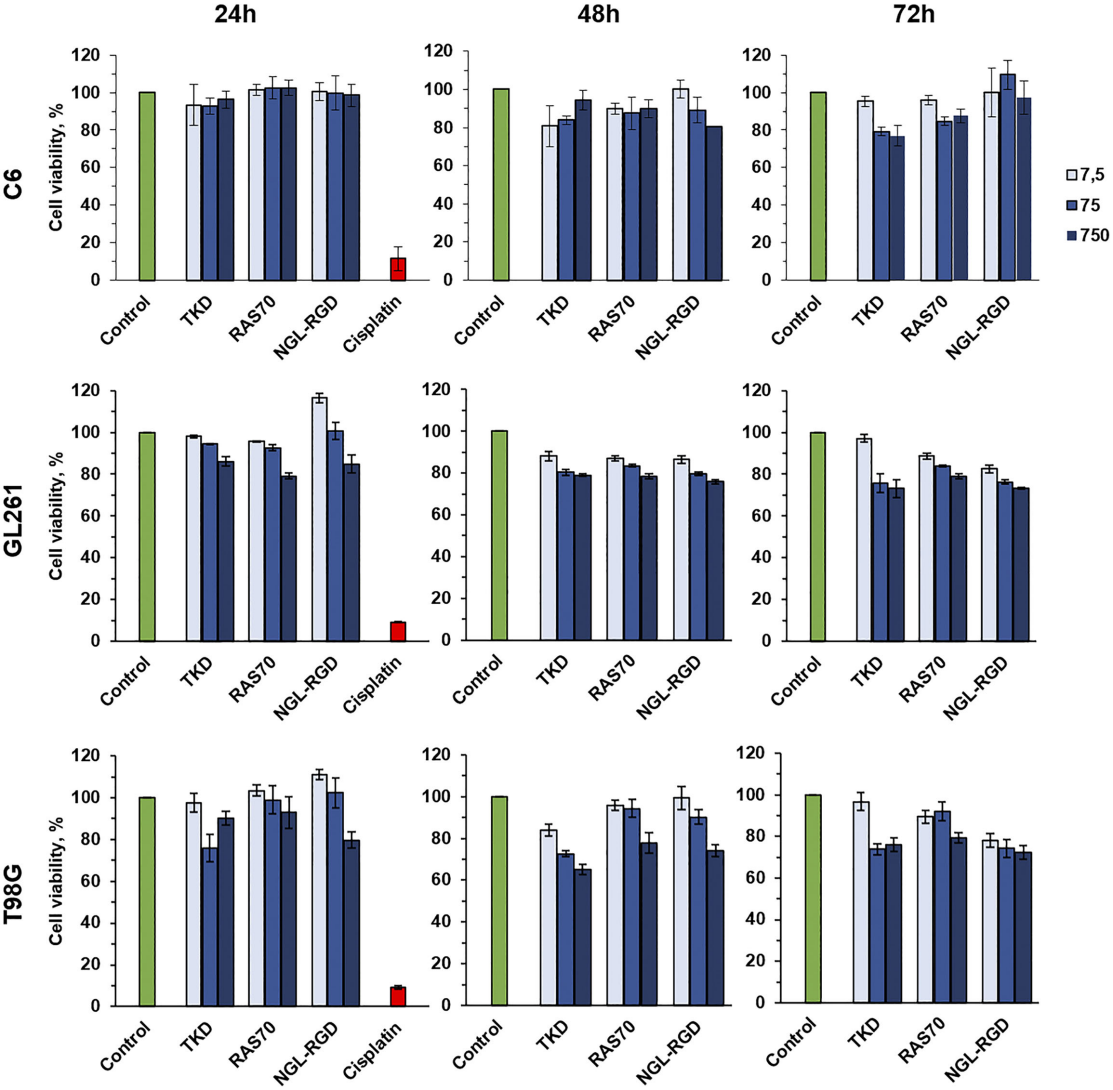
Cytotoxic effect analysis of the TKD, RAS70, NGL-RGD peptides using the MTT test at various peptides concentrations (7.5, 75, 750 µg/mL) and co-incubation times of 24, 48 and 72 hours. Cells without peptides were used as a control, cells incubated with 1 mg/mL of the cisplatin were used as a positive control.

Subsequently, we analyzed the cytotoxic effect of the peptides (RAS70, TKD, NGL, NGL-RGD) using the MTT test at various peptides concentrations (7.5, 75, 750 µg/mL) and co-incubation times of 24, 48 and 72 hours. We did not reveal the toxic effect of the fluorescently labeled peptides for all the cell lines studied (**Figure 7**, **Supplementary Table S1**).

**Figure 8:**
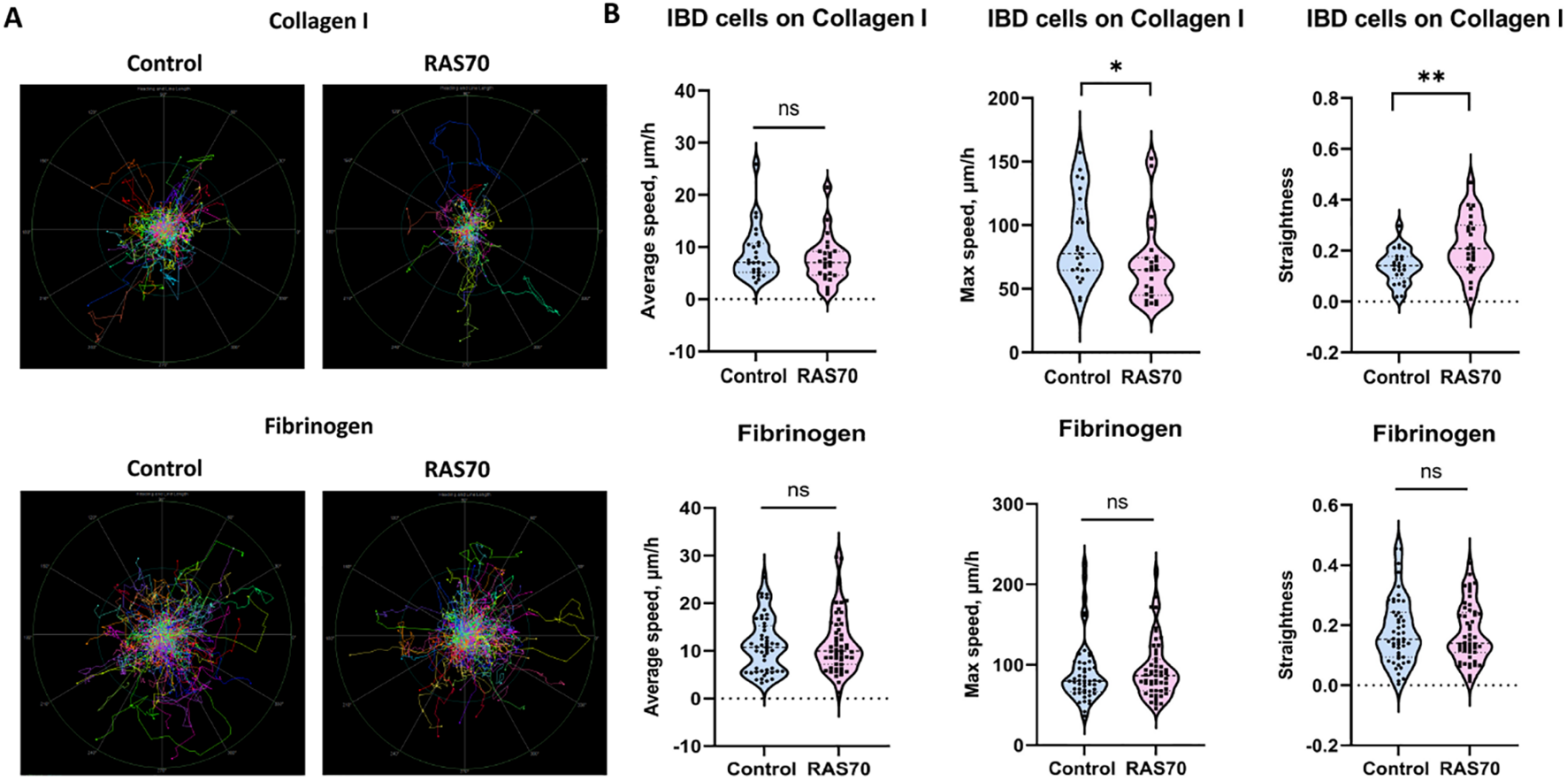
Motility analysis of patient-derived brain tumor primary glioblastoma cells IBD on various matrices (fibrinogen, collagen I) treated with RAS70 peptide. **(A)** Normalized (starting from the same starting point) tracks of motile cells (n = 50). **(B)** Violin plots of the motility metrics (average speed, max speed, and track straightness) calculated from tracks (n_tracks_ ≥ 294). Medians are indicated by the dashed horizontal line. Statistical differences are indicated at the 0.05 ( * ) and 0.01 ( ** ) level. ns, non-significant.

Additionally, we evaluated the effect of RAS70 peptide on cell motility of primary glioblastoma culture. In order to investigate the possible influence of the RAS70 peptide on the invasive capacity of tumor cells, we conducted an experiment in which we recorded cell motility on two different substrates (Collagen I, Fibrinogen) for 56 h. The results showed that for cells on collagen I, the peptide had no effect on the average cell speed, but slightly diminished maximum cell speed and increased the straightness of the trajectories. For cells on fibrinogen, the peptide did not affect any of the measured motility metrics (**Figure 8**).

### RAS70 peptide permeates the blood-brain barrier and accumulates in cancer cells

**Figure 9:**
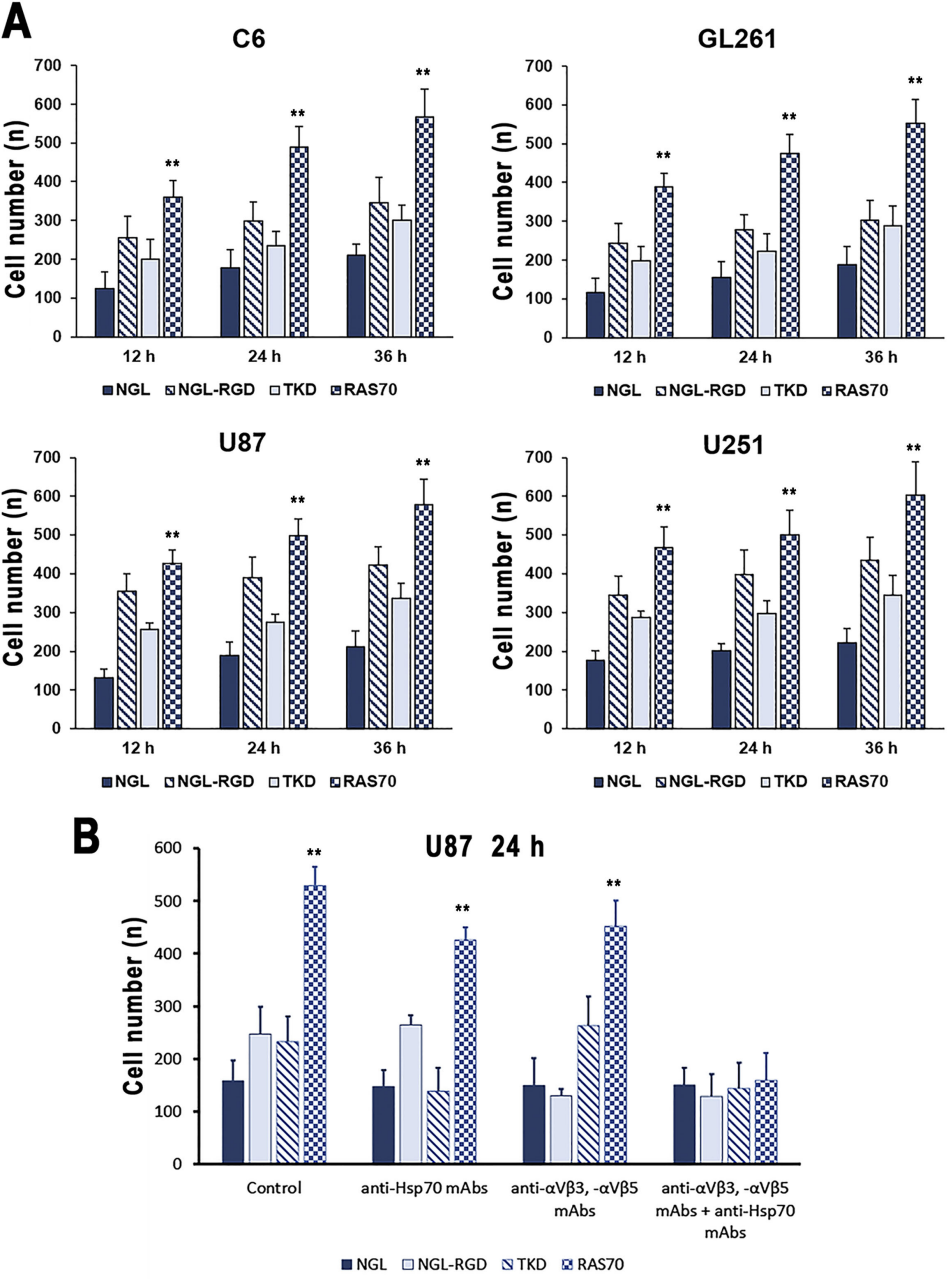
Transwell analysis of the accumulation of peptides labeled with the fluorescent dye in brain tumor cells. **(A)** C6, GL261, U87, and U251 cells were assessed in the lower chamber following adding of NGL, NGL-RGD, TKD, or RAS70 peptides in the upper chamber for 12, 24 and 36 hours. Data from 3 independent experiments are presented as M ± SE. **(B)** Assessment of mHsp70- and/or integrin (αvβ3, αvβ5)-mediated penetration of RAS70 into U87 cells in the transwell analysis. Prior to adding the peptides into upper chamber of transwell system U87 cells were pre-treated with anti-Hsp70 mAbs, anti- αvβ3 and -αvβ5 monoclonal antibodies or by combination of both. Data from 3 independent experiments are presented as M ± SE. ** p < 0.01 for testing mean expression levels.

To assess the penetrating properties of the peptides, a transwell system was used, where human umbilical vein endothelial cells (HUVEC) (5×10^5^ cells) and astrocytes (5×10^5^ cells) were seeded at the bottom of the upper chamber, and tumor cells (C6, GL261, U87, U251) (1×10^4^ cells) were seeded at the bottom of the lower chamber (33, 40). Then, a peptide labeled with a fluorescent dye (FITC) was introduced into the upper chamber and co-incubation was carried out for 12, 24 and 36 hours. At the end of the incubation, the tumor cells were washed with cold phosphate-buffered saline (PBS) and fixed with a 4% solution of paraformaldehyde in PBS. Nuclei were stained with 4’,6-diamidino-2-phenylindole (DAPI). (**Figure 9A**). According to the obtained data, it was shown that the TKD peptide penetrated the BBB, accumulating in tumor cells (within 36 hours of co-incubation) – 301 ± 35 (C6), 289 ± 51 (GL261), 337 ± 38 (U87), 345 ± 51 (U251) cells compared to the control scramble peptide (NGL) – 211 ± 29 (C6), 188 ± 47 (GL261), 211 ± 41 (U87), 221 ± 38 (U251) cells (P<0.05). The use of the RAS70 peptide resulted in a more pronounced passage of the peptide through the BBB and accumulation of the agent in tumor cells – 567 ± 72 (C6), 553 ± 61 (GL261), 578 ± 67 (U87), 603 ± 87 (U251) (P<0.05).

Next, we assessed the mHsp70- and integrin (αvβ3, αvβ5)-mediated penetration of the TKD and RAS70 via the BBB (**Figure 9B**). Thus, application of the anti-Hsp70 mAbs significantly reduced the penetration of both, TKD and RAS70, peptides but not the control peptides (NGL, NGL-RGD). When anti-αvβ3 and anti-αvβ5 mAbs were employed, we detected the decrease of NGL-RGD and RAS70 penetration via the BBB but not the NGL and TKD peptides. Combined treatment of cancer cells with anti-αvβ3 and anti-αvβ5 mAbs and anti-Hsp70 mAbs significantly suppressed the penetration of peptide (excluding the control peptide NGL) via the BBB and subsequent accumulation in cancer cells.

### Intravenous or local delivery of RAS70 results in the tumor-specific accumulation of the peptide in glioma models *in vivo*

**Figure 10:**
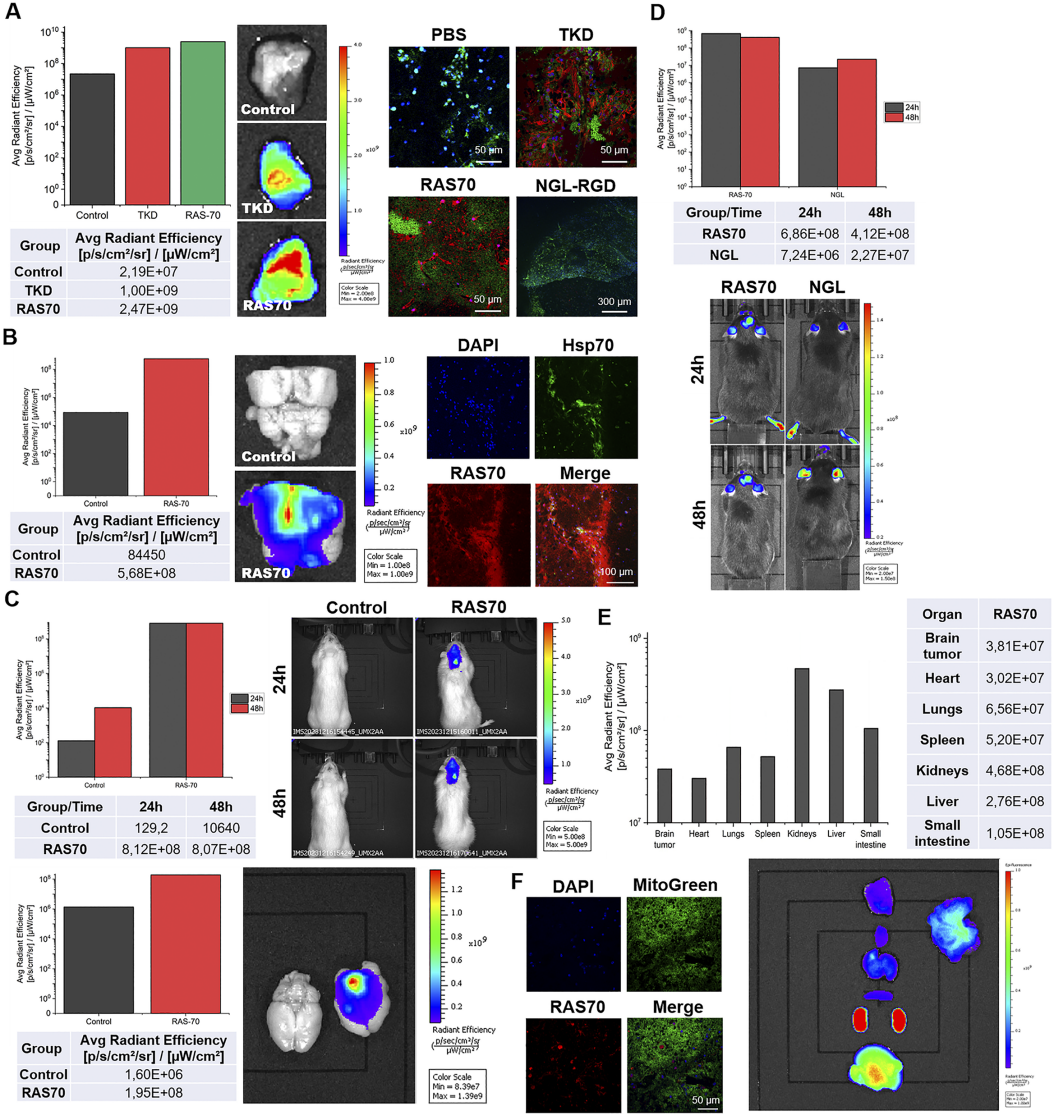
Tumor-specific accumulation of locally administered or intravenously injected RAS70 peptide in glioma-bearing animals. **(A)** Topical administration (exposure time of 5 min, 100 µg/mL) of the peptides in the GL261 glioma-bearing mice. Accumulation in the tissues was estimated using IVIS Spectrum imaging system. Retention of the peptides was further assessed employing live-cell inverted confocal microscopy when tissues were additionally stained for MitoGreen (green) and DAPI for nuclei (blue). Scale bars, 50 μm. **(B)** Topical administration (exposure 5 min, 100 µg/mL) of the peptides in the C6 glioma-bearing Wistar rats. Accumulation in the tissues was estimated using IVIS Spectrum imaging system. Retention of the peptides was further assessed employing live-cell inverted confocal microscopy when tissues were additionally stained for MitoGreen (green) and DAPI for nuclei (blue). Scale bars, 50 μm. **(C)** Evaluation of the RAS70 peptide accumulation in C6 glioma in rats 24 h following i.v. injection using IVIS Spectrum imaging system. Additionally, the freshly dissected brain was visualized for the RAS70 retention. **(D)** Evaluation of the RAS70 peptide accumulation in GL261 glioma in mice 24 h following i.v. injection using IVIS Spectrum imaging system with subsequent biodistribution analysis in various organs and tissues **(E)**. **(F)** representative confocal microscopy images of control mice brain (normal) co-stained for MitoGreen, nuclei (DAPI) and peptide RAS70. Scale bars, 50 μm.

Firstly, we evaluated retention of the RAS70 peptide when being topically sprayed over the freshly dissected tumor tissues. Thus, following 5 min of exposure to the RAS70 peptide we observed a significant increase of the detected signal over the tumor region of GL261 glioma in mice that was higher than the signal from the TKD peptide (**Figure 10A**).

The increased signal intensity was also observed when RAS70 peptide was used for targeting C6 rat glioma (**Figure 10B**). Subsequent histological analysis of the tumor samples employing live-cell inverted confocal microscopy imaging confirmed the accumulation of the targeted RAS70 peptide in the viable brain tumor cells that were also co-stained for Hsp70 expression (**Figures 10A, B**). Peptide was accumulating in the cell cytoplasm and in the endolysosomes (not penetrating the nuclei). When tissue sections were additionally stained for the mitochondria in order to evaluate the accumulation of the peptide in viable cells we detected retention of RAS70 in MitoGreen+ cells. Next, we assessed the accumulation of peptide in glioma-bearing animals following intravenous injection via the tail vein (**Figures 10C, D**). Already after 24 h we observed an accumulation of the RAS70 peptide in the brain tumors as compared to control. Subsequently, we evaluated the biodistribution of the i.v. administered RAS70 peptide in GL261 glioma-bearing mice (**Figure 10E**). As expected, apart from tumor accumulation we detected the highest retention of fluorescently-labeled agent in kidneys, liver and small intestine. When normal brain tissues we additionally assessed for the peptide we observed the non-specific presence of the RAS70 in blood vessel but not in normal brain cells (**Figure 10F**).

### RAS70 peptide accumulates in the mHsp70-positive brain tumors obtained from neuro-oncological patients

**Figure 11:**
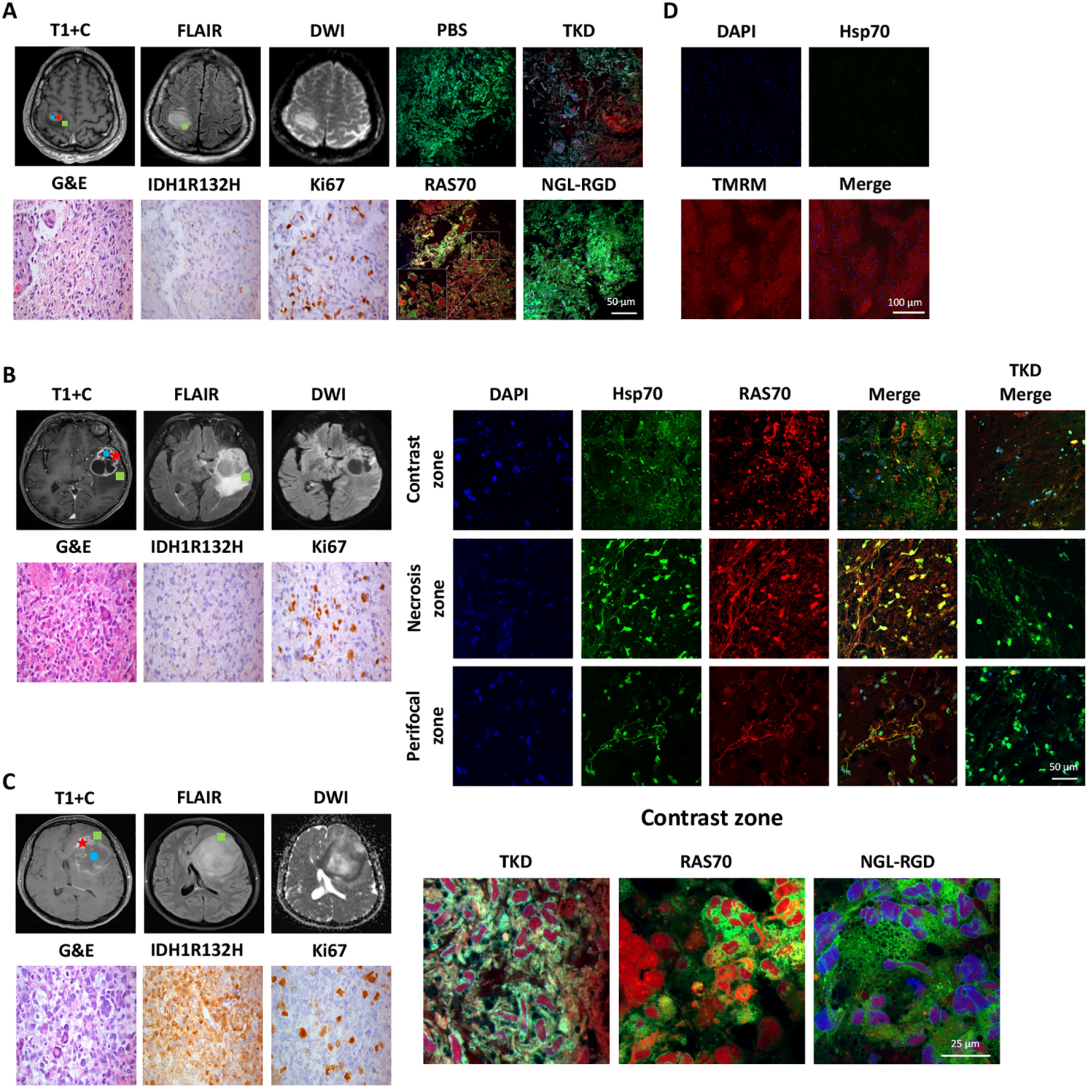
Evaluation of the RAS70 accumulation in brain tumor specimens by live-cell confocal microscopy imaging. Вased on preoperative MRI scans, surgical samples were selected from 3 distinct tumor regions for laboratory propagation. the green square - ≥ 3mm from enhancing margin and also within hyperintense FLAIR; the red star – contrast enhanced tumor zone; the blue circle - tumor сenter, hypointense on T1 and non-enhancing (necrotic zone). **(A)** Respective MR images (post-contrast T1-weighted, DWI, and Fluid-attenuated inversion recovery (FLAIR) images) of the patient BAV090467 are presented. Representative confocal microscopy images of the brain tumor samples stained for Hsp70 (red), MitoGreen (green). Nuclei were stained by DAPI. Scale bars, 100 μm. **(B)** Representative MR images (post-contrast T1-weighted, DWI, and FLAIR images) of the patient ZLS15051943 are presented. Tumor specimen (from three designated zones – contrast-enhancing, necrotic and perifocal) was additionally co-stained for Hsp70 by anti-Hsp70 FITC-labeled monoclonal antibodies (green). Nuclei were stained by DAPI. Scale bars, 100 μm. **(C)** Representative MR images (post-contrast T1-weighted, DWI, and FLAIR images) of the patient FSK28071987 are presented. Representative confocal microscopy images of the brain tumor samples stained for Hsp70 by RAS70 peptide (red), MitoGreen (green). Nuclei were stained by DAPI. Scale bars, 100 μm. **(D)** Representative confocal microscopy images of the normal brain tissue samples obtained from patient with epilepsy stained for Hsp70 (green) and TMRM (tetramethylrodamine methyl ester) (red). Nuclei were stained by DAPI (blue). Scale bars, 100 μm.

To study the target properties of the peptide RAS70 *ex vivo*, biopsy material was obtained from adult (n=6) and pediatric (n=2) neurooncological patients (**Table 1**). Tumor tissue was collected intraoperatively after preliminary determination of contrast-accumulating tissue according to MRI data, as described in (3). Subsequently, tumor was co-incubated with peptide at a concentration of 1 mg/mL for 5–10 minutes, after which the tissue sample was washed with a PBS solution and analyzed by live-cell inverted confocal microscopy (**Figures 11A–C**).

To detect peptide localization, the biopsy material was additionally stained with a mitochondrial marker MitoGreen and anti-Hsp70 antibodies (SPA-810). Accumulation of RAS70 was noted in all analyzed samples, with the peptide accumulating in viable cells that were also positive for the mitochondrial marker MitoGreen and Hsp70. A detailed analysis of the images showed that the RAS70 peptide was located in the same areas of the tumor tissue as the areas stained by anti-Hsp70 mAbs (**Figure 11**). Of note, when analyzing the necrotic areas, we also detected the non-specific retention of the peptide. Additionally, we assessed the depth of the RAS70-sCy3/sCy5 penetration into the tumor tissues which. For this, from the tumor samples following application of the peptides histological tissue sections were obtained with subsequent fluorescent microscopy assessment of the depth of the peptide penetration. For RAS70 peptide the depth constituted 3.0 ± 0.87 mm. When the peptide was used to stain normal brain, tissues obtained intraoperatively from patients with epilepsy (n=2), no tissue-specific localization of RAS70 was detected (**Figure 11D**).

## Discussion

The use of peptide drugs for precision diagnostics of malignant tumors, especially those with high infiltrative and metastatic activity, has become increasingly important in recent decades (41–43). In addition to the possibility of tumor detection using various fluorescent imaging systems, such peptide drugs allow for fairly accurate intraoperative determination of tumor and healthy tissue boundaries, which in turn allows for the most maximal tumor resection. In this study, the HSP70 protein was chosen as a target, which is associated not only with tumor cells with high invasive and metastatic activity, but also with GBM tumor stem cells (3). Given the presence of the BBB in brain tumors, which significantly affects the bioavailability of target drugs (especially in the perifocal tumor zone), the mHsp70-targeted peptide was conjugated with the RGD motif. The resulting peptide RAS70 had increased penetrative activity (**Figures 1**, **2**, **4**–**6**) compared to the TKD form of the peptide, which allowed the drug to accumulate effectively in glioma cells. Indeed, RAS70-sCy5 peptide accumulated in glioma cell lines as well as in primary GBM derived from pediatric and adult neurooncological patients. Subsequently, we quantified the pixel intensities and analyzed the colocalization of RAS70 and markers Rab5 and LAMP1 in tumor cells (**Figure 5**), showing the predominantly endolysosomal pathway of peptide internalization. Apart from microscopy image analysis intracellular pharmacokinetics (PK) could also be applied for quantification of peptide retention and distribution (44, 45).

Intravenous administration of the RAS70-sCy7.5 in orthotopic glioma model in animals resulted in the tumor retention of the peptide with a highest accumulation after 24 h (**Figure 10**). Subsequent histological studies showed the internalization of the peptide by glioma cells (that were also Hsp70-positive) (**Figure 10**). When tumor necrotic areas were assessed, we also observed non-specific accumulation of the peptide presumably due to the extravasation via the abnormal or impaired tumor vasculature. This vascular and non-specific signal enhancement should be taken into account when imaging hypervascularized (or with BBB damaged) histotypes of tumors.

Local spraying of the fluorescently labeled peptide resulted in highly effective tumor delineation of the freshly dissected brain tumor tissues in rodents (**Figure 10**). Recently, fluorescent-guided surgery has become increasingly important in tumor removal, particularly in glioblastoma resection (46). Indeed, several FDA-approved fluorescent agents, including 5-aminolevulinic acid (5-ALA) (47), fluorescein sodium (SF) (48, 49), and indocyanine green (ICG) (also known as second window indocyanine green (SWIG)) (50, 51) demonstrated potential in intraoperative tumor delineation. Currently, more targeting probes were designed based on nanoparticles, peptides, antibodies as well as chemical molecules (52, 53). Thus, Miller et al. reported application of the EGFR-targeting antibody conjugated with a fluorescent IR dye (cetuximab-IRDye800) in a Phase I clinical trial for imaging of contrast-enhancing glioblastoma (54). In another study by Patil et al., a tozuleristide (BLZ-100), tumor ligand chlorotoxin (CTX) conjugated to ICG, was employed for imaging newly diagnosed or recurrent gliomas (55). Peptide-based probes designed either to pass the BBB or target certain receptors on tumor cells also showed potential (56, 57). In our study the RAS-sCy7.5 peptide could not only cross the BBB but also to efficiently target mHsp70-positive tumor cells throughout the tumor mass, including the perifocal zone. In the current study we focused on the fluorescent imaging of the high-grade diffuse gliomas. Taking into account the presence of mHsp70 on the surface of various solid tumors (including gastric, colon, lower rectal carcinoma, and squamous cell carcinoma) topical application of RAS70 peptide could be potentially applied for fluorescent imaging of other tumor histotypes (58–61).

When biodistribution of the RAS70 was evaluated, we observed a high accumulation of the peptide in kidney > liver > small intestine > lungs (**Figure 10E**). The accumulation of the peptide in the following organs is most likely determined by the preferential routes of its elimination from the body (excretion via kidneys and partial excretion via the hepatobiliary pathway), which must subsequently be taken into account when conducting toxicological preclinical studies. Similar biodistribution was reported previously by Stangl et al. following i.v. administration of TPP-PEG_24_-DFO[^89^Zr] tracer in mice (12).

Subsequently, *ex vivo* analysis of the freshly resected tumors from neurooncological patients confirmed the accumulation of RAS70 in viable tumor cells (**Figure 11**). Following spraying over the normal brain and tumor tissues the exposition time constituted 5–10 minutes which underlines the convenient application of the peptide in the intraoperative fluorescence-guided tumor resection, particularly when being compared to the conventional fluorescent agents. When compared, 5-ALA agent has to be orally administered at a dose of 20 mg/kg body weight at least 3 h prior to surgery (62), ICG has to be i.v. administered at a dose of 5 mg/kg 24 h before surgery (63), and SF upon completion of anesthesia procedures could be i.v. injected at a dosage of 5 mg/kg (48). The use of the peptide RAS70 is possible by a neurosurgeon directly during the operation, depending on the need to visualize malignant tissue, which significantly expands the maneuverability of actions compared to the above-described drugs that require intravenous administration prior to the tumor removal. Another advantage is the possibility of multiple application of the peptide to tissues throughout the operation without being tied to a specific time frame for the best effectiveness of the drug (thus for 5-ALA the recommended timeframe constitutes 2–4 hours). In our experimental setting for *ex vivo* imaging fluorescently-labelled RAS70 could penetrate the glioblastoma tissues after topical application on the depth of 3.0 ± 0.87 mm, indicating the necessity of repeated application of the peptide in the clinical settings. In our study the reported depth of RAS70 penetration into tissues was significantly higher as compared the Hsp70-targeting peptide without RGD motif (0.72 ± 0.03 mm) that was recently demonstrated by Holzmann et al. (25). Presumably, inclusion of the tripeptide into the RAS70 formula increased the tumor-penetrating properties of the latter.

GBM-homing properties of the RAS70 peptide could be further exploited for the delivery of anti-tumor agents including radionuclides, chemotherapeutic drugs, etc. Thus, in a recent study Wu et al. reported that mHsp70-targeting peptide coupled with hybrid iron oxide (Fe_3_O_4_)-gold (Au) nanoparticles (FeAuNPs) facilitated accumulation of particles in breast cancer cells that was further used for the cells radiosensitization upon ionizing irradiation (22).

An *in vitro* experiment with the effect of the RAS70 peptide on the motility of glioblastoma cells demonstrated that the invasiveness of cells does not increase, although it was possible to notice a change in the shape of trajectories for cells on Collagen I. It is known that Hsp70 in cancer cells can be localized in membrane lipid rafts, and such key regulators of cell motility as small GTPases of the Rho family were also found there (64). It is possible that by interfering in membrane rafts with membrane-associated Hsp70, the peptide causes changes in signaling pathways associated with small GTPases, which leads to changes in the nature of cell motility. This assumption is also supported by the fact that application of Hsp70 inhibitors decreases the cancer cell lines migratory activity *in vitro* (3).

Since chemo-radiation therapy also acts as a cell stressor, and can elevate the expression of heat shock proteins, including HSP70, in brain tumors mHsp70-targeting with RAS70 peptide can be combined with other radio-immunotherapies to potentiate their effect. Previously, Fellinger et al. demonstrated the kinetics of the mHsp70 expression on the U87 human glioblastoma cells following irradiation in order to identify the therapeutic window for Hsp70 targeting (65). Already at dose of 2 Gy the authors observed an upregulated mHsp70 density on the membrane U87 cells which peaked on day 4 and subsequently declined on day 7. Indeed, when Hsp70-targeted superparamagnetic nanoparticles (SPIONs) were combined with brain tumor irradiation the highest accumulation of the agents was detected in animals (11, 15). Presumably, RAS70 targeting can be also combined not only with radio- but with other methods of tumor immunotherapies (66, 67) including immunomodulatory and/or glioma-targeting peptides (68).

In conclusion, mHsp70-targeted RAS70-sCy7.5 peptide demonstrated high glioma-homing properties (when being intravenously administered) efficiently crossing the blood-tumor barrier and accumulating in the tumor cells in contrast-enhancing and perifocal areas. Local delivery of the peptide over the dissected normal brain and glioma tissues facilitated the delineation of the tumor via employing fluorescent visualization. Taking into account the low cost, high efficiency in tumor tissue penetration and internalization by cancer cells, convenient application (spraying over the area of interest with a short exposition time of 5–10 min), absence of cytotoxicity (as shown by MTT assay) makes RAS70 a potential agent for further investigation in clinical trials of fluorescence-guided tumor surgery.

## Data Availability

The raw data supporting the conclusions of this article will be made available by the authors, without undue reservation.
