# Supplementary material for: RAS70 peptide targets multiforme glioblastoma by binding to the plasma membrane heat shock protein HSP70
Source: Front Oncol. 2025 Mar 24;15:1543657. doi: 10.3389/fonc.2025.1543657 (PMC11973282; doi:10.3389/fonc.2025.1543657)
Supplement: Supplementary file 1 [file DataSheet1.pdf]

Supplementary material

RAS70 peptide targets multiforme glioblastoma by binding to the plasma membrane heat shock protein HSP70

Maxim Shevtsov[1–3]*, Natalia Yudintceva[2,3], Danila Bobkov[2–4], Ruslana Likhomanova[2,3], Anastasiya Nechaeva[2], Elena Mikhailova[2], Elena Oganesyan[2], Viacheslav Fedorov[2], Andrey Kurkin[3], Anastasiya Lukacheva[2,3], Aleksander Kim[2], Evegeniy Fedorov[2], Daria Sitovskaya[5], Alexey Ulitin[5], Natalia Mikhailova[2], Ilya Anufriev[2], Maria Istomina[2], Ekaterina Murashko[2], Elizaveta Kessenikh[2], Nikolay Aksenov[3], Yulia Vakhitova[2], Konstantin Samochernykh[2,5], Emil Pitkin[6], Evgeny Shlyakhto[2], Stephanie E. Combs[1]

[1]Department of Radiation Oncology, Klinikum rechts der Isar, Technical University of Munich, 81675 Munich, Germany

[2]Personalized Medicine Centre, Almazov National Medical Research Centre, Akkuratova Str. 2, 197341 St. Petersburg, Russia

[3]Laboratory of Biomedical Nanotechnologies, Institute of Cytology of the Russian Academy of Sciences (RAS), 194064 St. Petersburg, Russia

[4]Smorodintsev Research Institute of Influenza, Professora Popova str, 15/17, 197376 St. Petersburg, Russia

[5]Polenov Neurosurgical Institute, Almazov National Medical Research Centre, Mayakovskogo st. 12, 191014 St. Petersburg, Russia;

[6]Department of Statistics and Data Science, Wharton School, University of Pennsylvania, Philadelphia, 19104 PA, United States

*** Correspondence:**

Dr. Maxim Shevtsov

Tel.: +49-173-1488882

maxim.shevtsov@tum.de

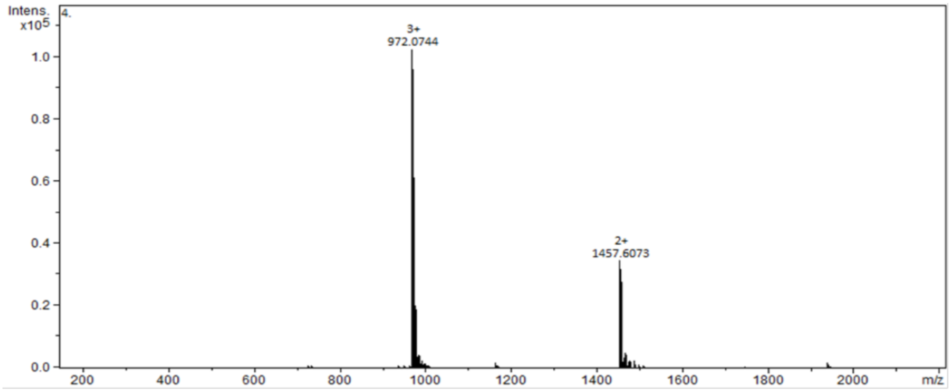

Supplementary Figure S1. HPLC-QTOF-MS mass spectra for RAS70 ($C_{127}H_{182}N_{29}O_{42}S_4$).

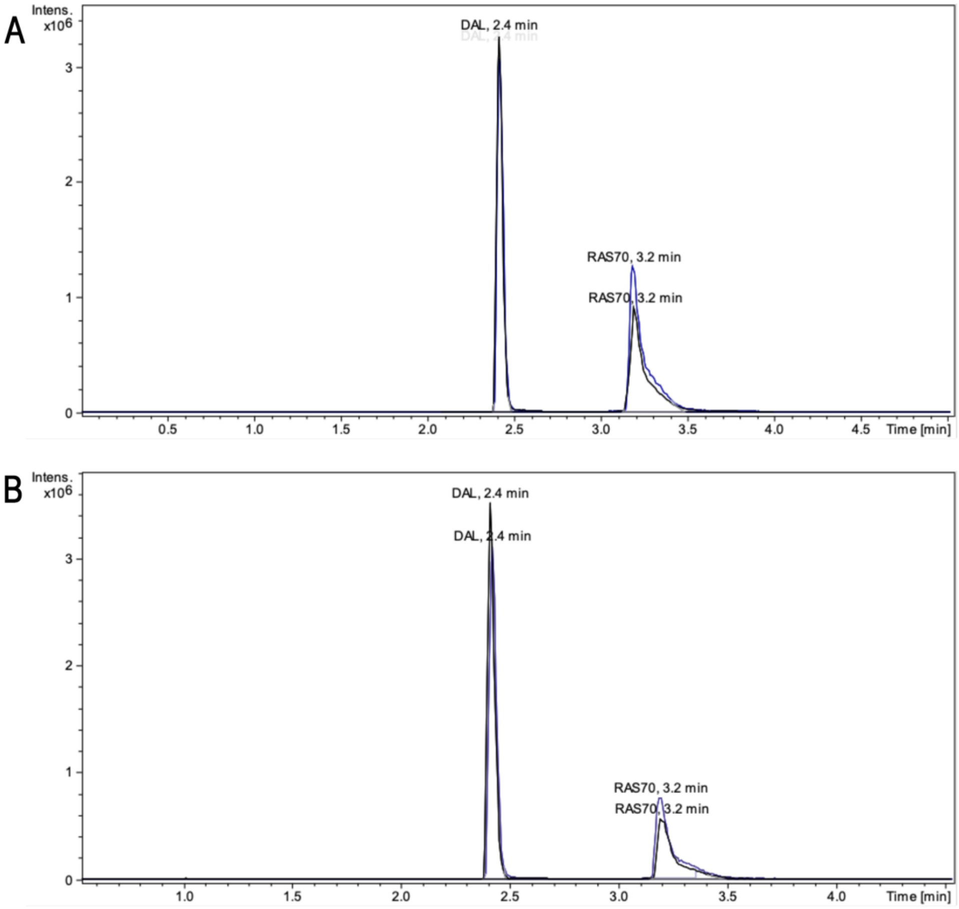

Supplementary Figure S2. Extracted ion chromatogram (EIC) of LC-QTOF-MS analysis of freshly prepared (blue) working solution of RAS70 and the working solution after 1 week (black) of storage at +4 °C **(A)** and RT **(B)** in Protein LoBind Tubes: DAL (Dalargin, C35H51N9O8 $[M+H]^+$, 726.3933±0.005 m/z); TKD2-sCy7.5 (RAS70, C127H182N29O42S4 $[M+H]^{3+}$, 972.0699±0.005 m/z).

Cell line	Peptide	Concentration, µg/mL	Incubation time, h					
			24		48		72	
			sample size	mean±SD, %	sample size	mean±SD, %	sample size	mean±SD, %
C6	w/o, control	-	21	100	24	100	15	100
	TKD	7.5	12	93.41±5.64	12	80.82±10.83	12	95.40±2.65
		75	12	92.78±4.13	12	83.84±2.34	12	79.28±2.37
		750	12	96.30±4.5	12	94.30±4.11	12	76.98±5.45
	RAS70	7.5	12	101.46±8.36	12	89.99±2.85	12	95.96±2.64
		75	12	102.38±5.86	12	87.42±8.46	12	84.70±2.28
		750	12	102.54±4.18	12	89.89±5.30	12	87.71±3.65
	NGL-RGD	7.5	21	100.45±07.30	23	100.00±4.72	20	100.02±12.85
		75	21	99.76±9.10	24	89.14±6.79	9	109.37±7.82
		750	18	98.52±5.86	21	80.52±4.65	19	97.22±8.78
	Cisplatin	1000	9	11.56±6.50	-	-	-	-
GL261	w/o, control	-	12	100	12	100	12	100
	TKD	7.5	8	98.12±0.67	8	88.07±02.17	8	97.10±1.92
		75	8	94.31±0.38	8	80.27±1.61	8	75.82±4.44
		750	8	86.03±2.27	8	78.95±0.66	8	73.14±4.39
	RAS70	7.5	8	95.53±0.40	8	87.03±1.34	8	88.61±1.47
		75	8	92.58±1.39	8	83.52±0.66	8	83.79±0.48
		750	8	78.98±1.42	8	78.50±1.11	8	78.93±1.36
	NGL-RGD	7.5	8	116.53±2.29	8	86.44±1.92	8	82.52±1.95
		75	8	100.62±3.91	8	79.80±0.77	8	76.22±1.00
		750	8	84.75±4.29	8	75.94±0.98	8	73.28±0.54
	Cisplatin	1000	12	9.17±0.15	-	-	-	-
T98G	w/o, control	-	11	100	12	100	12	100
	TKD	7.5	11	97.50±4.41	12	83.91±2.91	12	96.68±4.23
		75	12	75.72±6.54	12	72.58±1.48	12	73.77±2.79
		750	12	90.10±3.25	12	65.13±2.54	12	76.02±3.48
	RAS70	7.5	12	103.42±2.64	12	95.71±2.51	12	89.46±3.18
		75	12	98.92±6.75	12	94.37±4.30	12	92.01±4.48
		750	12	93.05±7.57	12	77.85±4.97	12	79.40±2.49
	NGL-RGD	7.5	12	111.23±2.46	12	99.39±5.65	12	78.09±3.33
		75	12	102.31±7.15	12	90.24±3.58	11	74.19±4.17
		750	11	79.63±3.85	11	74.31±2.83	10	72.18±3.28
	Cisplatin	1000	12	9.17±0.70	-	-	-	-

Supplementary Table S1. MTT-assay of cell viability, %.
